# A general framework for optimizing arterial spin labeling MRI experiments

**DOI:** 10.1002/mrm.27580

**Published:** 2018-12-26

**Authors:** Joseph G. Woods, Michael A. Chappell, Thomas W. Okell

**Affiliations:** ^1^ Wellcome Centre for Integrative Neuroimaging, FMRIB, Nuffield Department of Clinical Neurosciences University of Oxford Oxford United Kingdom; ^2^ Institute of Biomedical Engineering, Department of Engineering University of Oxford Oxford United Kingdom

**Keywords:** accuracy, arterial spin labeling, cerebral blood flow, multi‐delay, optimal experimental design, perfusion

## Abstract

**Purpose:**

Arterial spin labeling (ASL) MRI is a non‐invasive perfusion imaging technique that is inherently SNR limited, so scan protocols ideally need to be rigorously optimized to provide the most accurate measurements. A general framework is presented for optimizing ASL experiments to achieve optimal accuracy for perfusion estimates and, if required, other hemodynamic parameters, within a fixed scan time. The effectiveness of this framework is then demonstrated by optimizing the post‐labeling delays (PLDs) of a multi‐PLD pseudo‐continuous ASL experiment and validating the improvement using simulations and in vivo data.

**Theory and Methods:**

A simple framework is proposed based on the use of the Cramér‐Rao lower bound to find the protocol design which minimizes the predicted parameter estimation errors. Protocols were optimized for cerebral blood flow (CBF) accuracy or both CBF and arterial transit time (ATT) accuracy and compared to a conventional multi‐PLD protocol, with evenly spaced PLDs, and a single‐PLD protocol, using simulations and in vivo experiments in healthy volunteers.

**Results:**

Simulations and in vivo data agreed extremely well with the predicted performance of all protocols. For the in vivo experiments, optimizing for just CBF resulted in a 48% and 15% decrease in CBF errors, relative to the reference multi‐PLD and single‐PLD protocols, respectively. Optimizing for both CBF and ATT reduced CBF errors by 37%, without a reduction in ATT accuracy, relative to the reference multi‐PLD protocol.

**Conclusion:**

The presented framework can effectively design ASL experiments to minimize measurement errors based on the requirements of the scan.

## 1 INTRODUCTION

Arterial spin labeling (ASL) is a non‐invasive MRI technique that can be used to quantify brain tissue perfusion.^1^, ^2^ Blood water entering the brain is labeled by magnetic inversion and, after a post‐labeling delay (PLD), an image is acquired. The PLD gives the labeled blood time to travel from the labeling region to the tissue bed,^3^ this time being referred to as the arterial transit time (ATT). This labeled image is subtracted from a control image, where the blood is not labeled, resulting in an image with the signal intensity proportional to cerebral blood flow (CBF), assuming the complete arrival of labeled blood water. A model inversion then allows for CBF quantification.

It is common to use a single PLD,^4^ which allows many averages to be acquired within a short scan time, therefore increasing SNR. However, if the PLD is shorter than the ATT, CBF can be severely underestimated. The use of longer PLDs reduces the risk of such errors but this must be balanced against the loss of tracer signal through T_1_ decay and increased noise because of fewer averages being achievable within a given scan time.

An alternative method is to use multiple PLDs and to fit a kinetic signal model to the resulting dynamic data.^5^ In this way, both CBF and ATT can be estimated, reducing the bias caused by unknown ATT and also providing extra, potentially clinically useful, information.^6^ However, multiple PLDs present a more complicated experimental design problem: when should the ASL signal be sampled to give the most accurate CBF and ATT measurements? This increased complexity, along with a more involved analysis process, has restricted the use of multi‐PLD techniques.^4^ Studies that do use multiple PLDs often use equally spaced PLDs over a range of times reflective of the expected ATTs and based on previous experience.^7^, ^8^

The field of optimal experimental design provides a mathematical framework, the Cramér‐Rao lower bound (CRLB),^9^, ^10^ with which to design experiments to minimize the variance of estimated parameters. Previous studies, which used the CRLB to optimize the inversion time (TI) in PASL experiments, have shown promise, but were limited by: (1) aggregating a series of locally optimal TIs,^11^, ^12^ (2) optimizing across both ATT and CBF prior distributions,^12^, ^13^ and (3) use of Gaussian prior distributions over the parameter values.^11^, ^12^, ^13^ Kramme et al.^14^ also proposed increasing the number of averages at longer PLDs to improve CBF accuracy, but this method does not directly consider the parameter uncertainty.

In this work, we present a flexible framework for designing ASL protocols by maximizing the accuracy of CBF, or both CBF and ATT, estimates. We build on previous studies^11^, ^13^ to produce a simplified framework that combines the information obtained across all acquisitions, optimizing the experiment as a whole, within a predefined scan time. Furthermore, we demonstrate that an a priori CBF distribution is not required. This flexible and simplified optimization framework is intended to improve the accessibility and use of optimal experimental design for ASL experiments.

We demonstrate the effectiveness of this framework by optimizing the PLDs for a multi‐PLD pseudo‐continuous ASL^15^ (PCASL) experiment, using a 2D multi‐slice readout across a uniform ATT distribution appropriate for gray matter (GM) in healthy volunteers. We generate 2 protocols: one which minimizes both CBF and ATT errors, and one which minimizes only CBF errors while remaining insensitive to ATT variation. We compare these protocols against matched scan‐time reference multi‐PLD^16^ and single‐PLD protocols, using Monte Carlo simulations and in vivo experiments, and demonstrate that the use of a broad, population‐specific, uniform ATT distribution can successfully reduce CBF and ATT errors. This study builds on previously presented work.^17^

## 2 THEORY

### 2.1 Cramér‐Rao lower bound

The Cramér‐Rao lower bound^9^, ^10^ provides a mathematical expression for the lower bound on the variance (uncertainty) of parameters estimated from a set of data. More specifically, it states that the inverse of the Fisher information matrix (FIM) is the lower bound on the covariance matrix for deterministic parameters:

(1) $$F^{-1}\leq cov\left(\theta \right),$$

where $F^{-1}$ is the inverse of the FIM and cov(θ) is the covariance matrix for a vector of model parameters, θ.

For ASL experiments, the Fisher information matrix takes the form:

(2) $$F{\left(t;\theta \right)}_{\mathrm{jk}}=\frac{A}{\sigma^{2}}\sum_{i=1}^{N}\frac{\partial \Delta M\left(t_{i};\theta ;\rho \right)}{\partial \theta_{j}}\frac{\partial \Delta M\left(t_{i};\theta ;\rho \right)}{\partial \theta_{k}},$$

where *N* is the number of acquisitions, *A* is the integer number of averages for each acquisition achievable in a given scan time, $\sigma^{2}$ is the normally distributed noise variance of the acquired images, ΔM is the ASL difference signal model, t is a vector containing the experimental timings, θ is a vector of the model parameters to be inferred, ρ are the remaining model parameters which are assumed fixed, and $\frac{\partial \Delta M}{\partial \theta }$ are the sensitivity functions of the signal model. Because it is common to assume known values for all model parameters except CBF and ATT, here we set θ = [CBF, ATT] and drop the explicit reference to ρ for simplicity.

Note that we have assumed identical noise variance across acquisitions, unlike Xie et al,^11^ where the authors found an empirical noise model across acquisitions. This simplification means that the noise variance is inversely proportional to the number of averages for the assumed case of normally distributed noise. An optimal protocol can then be generated within a fixed scan time by calculating the number of averages possible for a given design within this time. A known value for the noise variance is not required for optimization because it is only a scaling factor, however, knowledge of this value will generate CRLB variances in physiological units. This also means that the optimization will be equally valid for all voxels in images with spatially varying SNR, such as when a head array coil is used for signal detection.

### 2.2 Optimization criteria

The most widely used optimality criterion is the determinant of the covariance matrix. Because the determinant of a matrix is equal to the product of its eigenvalues, it is proportional to the volume of the confidence ellipsoid of the estimated parameters. By minimizing the determinant, we minimize the volume of this confidence ellipsoid. We can define this D‐optimality criterion as:

(3) $$\begin{array}{c} \underset{t}{\min}\;{\phi \left(t;\;\theta \right)}_{D-optimal}=\underset{t}{\min}\;det\left(cov\left(t;\;\theta \right)\right)\\ =\min_{t}\frac{1}{\det\left(F\left(t;\;\theta \right)\right)} \end{array},$$

because $1/\det\left(A\right)=det\left(B\right)$ when $A=B^{-1}$.

The second optimality criterion explored in this study is the minimization of only the CBF variance, because CBF is often the main parameter of interest, with ATT being a confounding parameter. Conceptually, this objective function aims to minimize CBF variance, including minimization of the impact of ATT on CBF measurements. This criterion, referred to as L‐optimality, is given by:

(4) $$\min_{t}\;{\phi \left(t;\;\theta \right)}_{L-optimal}=\min_{t}\;trace\left(W{F\left(t;\;\theta \right)}^{-1}\right),$$

where W is a symmetric positive semi‐definite matrix and has the same dimensions as F.^18^ In this case, $W_{11}$ is the only non‐zero element, to select only the CBF variance.

### 2.3 (P)CASL signal model

In this study, we use the general kinetic model^5^ for (P)CASL:

(5) $$\begin{array}{ccc} \Delta M\left(t\right) & =0 & 0<t<\Delta t\\ & =2M_{0B}fT_{1}^{\prime }\alpha e^{\frac{-\Delta t}{T_{1b}}}\left(1-e^{\frac{-(t-\Delta t)}{T_{1}^{\prime }}}\right) & \Delta t<t<\tau +\Delta t\\ & =2M_{0B}fT_{1}^{\prime }\alpha e^{\frac{-\Delta t}{T_{1b}}}e^{\frac{-(t-\tau -\Delta t)}{T_{1}^{\prime }}}\left(1-e^{\frac{-\tau }{T_{1}^{\prime }}}\right) & \tau +\Delta t<t \end{array},$$

where$1/T_{1}^{\prime }=1/T_{1t}+f/\lambda$, ΔM is the ASL signal difference between label and control images, t is the time from the start of labeling (s), $M_{0B}$ is the equilibrium magnetization of blood, f is the CBF (s^−1^), λ is the equilibrium brain–blood water partition coefficient (mL g^−1^), α is the labeling efficiency, Δt is the arterial transit time (s), τ is the labeling duration (s), and $T_{1b}$ and $T_{1t}$ are the longitudinal relaxation time constants for arterial blood and tissue (s), respectively. Note that any appropriate analytical signal model may be used instead.

### 2.4 Sensitivity functions

To simplify the CBF sensitivity function, we assume that the apparent tissue relaxation time, $T_{1}^{\prime }$, is fixed with respect to f (fixed outflow), as proposed by Xie et al.^11^ Using this assumption, with outflow at 50 ml/100g/min, leads to a maximum error of ~1.78% in the simplified CBF sensitivity function, when the true value of f is in the range 0 – 100 ml/100g/min. The sensitivity functions are then:

(6) $$\begin{array}{ccc} \frac{\partial \Delta M\left(t\right)}{\partial f} & =0 & 0<t<\Delta t\\ & =2M_{0B}T_{1}^{\prime }\alpha e^{\frac{-\Delta t}{T_{1b}}}\left(1-e^{\frac{-(t-\Delta t)}{T_{1}^{\prime }}}\right) & \Delta t<t<\tau +\Delta t\\ & =2M_{0B}T_{1}^{\prime }\alpha e^{\frac{-\Delta t}{T_{1b}}}e^{\frac{-(t-\tau -\Delta t)}{T_{1}^{\prime }}}\left(1-e^{\frac{-\tau }{T_{1}^{\prime }}}\right) & \tau +\Delta t<t \end{array},$$

(7) $$\begin{array}{ccc} \frac{\partial \Delta M\left(t\right)}{\partial \Delta t} & =0 & 0<t<\Delta t\\ & =2M_{0B}fT_{1}^{\prime }\alpha e^{\frac{-\Delta t}{T_{1b}}}\left[-\frac{1}{T_{1b}}-e^{\frac{-\left(t-\Delta t\right)}{T_{1}^{\prime }}}\left(\frac{1}{T_{1}^{\prime }}-\frac{1}{T_{1b}}\right)\right] & \Delta t<t<\tau +\Delta t\\ & =2M_{0B}fT_{1}^{\prime }\alpha e^{\frac{-\Delta t}{T_{1b}}}e^{\frac{-(t-\tau -\Delta t)}{T_{1}^{\prime }}}\left(1-e^{\frac{-\tau }{T_{1}^{\prime }}}\right)\left(\frac{1}{T_{1}^{\prime }}-\frac{1}{T_{1b}}\right) & \tau +\Delta t<t \end{array}.$$

**Figure 1:**
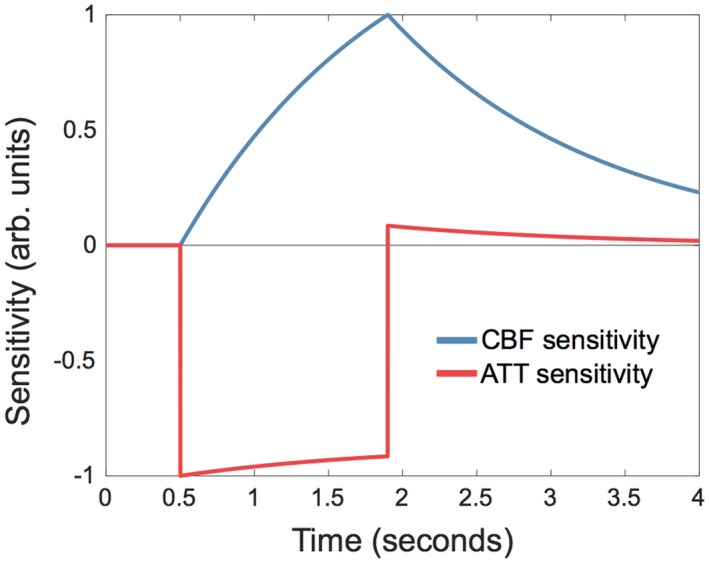
Example of normalized sensitivity functions for CBF (blue) and ATT (red). Parameters used: labeling duration = 1.4 s; CBF = 50 mL/100 g/min; ATT = 0.5 s; *T*_1*b*_ = 1.65 s; *T*_1*t*_ = 1.445 s; λ = 0.9

Shown in Figure 1, they demonstrate that the model is most sensitive to CBF when the signal is maximized (t=τ+Δt) and most sensitive to ATT during inflow (Δt<t<τ+Δt).

The complete CBF sensitivity function, where $T_{1}^{\prime }$ is dependent on f, is given in Supporting Information Text S1. The equivalent sensitivity functions for PASL may be found in Xie et al.^11^

### 2.5 Priors

The sensitivity functions depend on the parameters being estimated: CBF and ATT. A protocol that minimizes the CRLB at single set CBF and ATT values (locally optimal), will be a poor choice at values far from these.^18^, ^19^ A priori information of the likely range of parameter values is therefore required to minimize the estimator variance over them.

We use the method proposed by Gilmour et al.,^18^ referred to as average or pseudo‐Bayesian optimal design. Here, the optimality criterion, $\phi \left(t;\theta \right)$, is averaged across a prior probability distribution, $p\left(\theta \right)$, and minimized. Therefore, the optimization problem is:

(8) $$\min_{t}\Psi \left(t;\theta \right)=\min_{t}E\left[\phi \left(t;\theta \right)\right]=\min_{t}\int_{\theta }\phi \left(t;\theta \right)p\left(\theta \right)d\theta .$$

This integral is difficult to evaluate analytically, so we approximate it with a number of equally spaced samples from the prior distribution:

(9) $$\tilde{\Psi }\left(t\right)=\frac{1}{r}\sum_{l=1}^{r}\phi \left(t;\theta =\theta_{l}\right)p\left(\theta =\theta_{l}\right)≃\Psi \left(t;\theta \right),$$

where $\theta_{l}$ is a sample of parameters from the prior distribution, and r is the number of samples.

In previous work, both CBF and ATT prior probability distributions were used.^11^, ^12^, ^13^ However, using Equations (6) and (7), we note that $\phi_{L-optimal}$ does not depend on CBF, and $\phi_{D-optimal}$ simply scales with CBF, therefore, the optimal protocol generated using each optimality criterion will be identical for any value of CBF (see Supporting Information Text S2 and Supporting Information Figure S1). Therefore, the optimal design will only depend on the ATT distribution, which greatly reduces the required prior knowledge and the number of calculations involved in the optimization, because a point prior may be used for CBF.

## 3 METHODS

All optimizations, simulations and analysis were performed using MATLAB (The MathWorks, Natick, MA).

### 3.1 PLD optimization

The optimization theory introduced above is general and can be used to optimize any type of ASL experiment for parameter inference. Here, we describe the specific methods that we employed for optimizing a 2D‐EPI multi‐slice, multi‐PLD, fixed label duration PCASL experiment, to find the optimal set of PLDs for CBF and ATT estimation.

**Figure 2:**
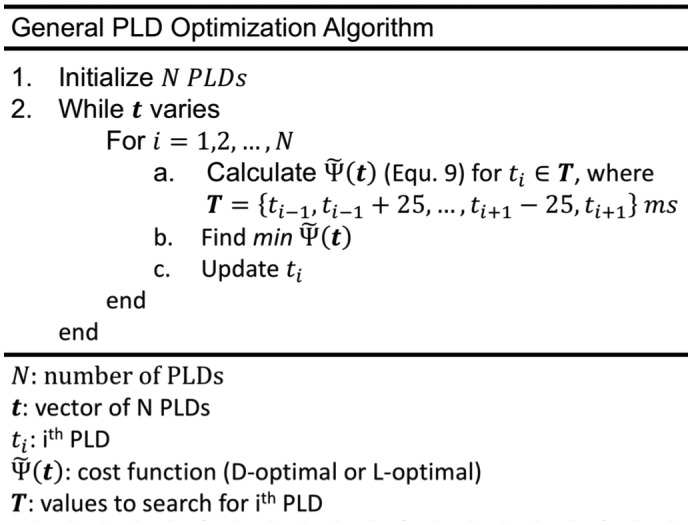
Pseudocode outlining the PLD optimization algorithm used in this study. The optimal number of PLDs, *N*, can be found by running this algorithm for a range of *N* and finding the design which minimizes Equation 9

Two protocols, referred to as CBF‐ATT_opt_ and CBF_opt_, were generated using the optimality criteria in Equations (3) and (4) (D‐optimal and L‐optimal), respectively. The optimization algorithm developed for this study uses an iterative exchange method similar to Xie et al.^11^ and aims to find the optimal set of PLDs that minimizes Equation 9. The pseudocode outline is shown in Figure 2. It takes as inputs the number of PLDs, ATT prior probability distribution, available scan duration, number of slices, slice duration, and the remaining constants in Equations 6 and 7. The PLDs were initialized to be equally distributed in the interval 0.25≤PLD≤1.5 s. The algorithm loops through the PLDs, selecting the optimal PLD at each step from a list of possible values. The PLD list for t_i_ (the i^th^ PLD) was restricted to the interval $0.2s\;{\leq t}_{i-1}\leq t_{i}\leq t_{i+1}\leq 3$ s, using 25‐ms increments. This reduces the size of the search without restricting the algorithm from finding the approximate optimal solution. The minimum PLD of 0.2 s was to provide enough time for the BGS inversion pulses used in vivo. The longest PLD chosen by the algorithm during testing was always at most equal to the longest ATT in the ATT prior probability distribution. The PLD upper bound of 3 s will, therefore, not restrict the choice of PLDs in this study (see below), but will reduce the PLD search space.

An ATT range of 0.5≤ATT≤1.8 s was assumed for GM in healthy volunteers.^4^, ^20^ A uniform prior probability distribution for this range was used to ensure equal weighting of all ATTs. To avoid edge effects, the ATT distribution was extended on either side by 0.3 s with linearly decreasing probability. The CBF point prior was set to 50 mL/100 g/min. The readout duration was assumed to be 1.275 s to give realistic scan times. The total allowable scan time was set to 5 min. Variable TR was assumed such that a short PLD is acquired with a correspondingly short TR to minimize dead time in the sequence.^21^ The number of averages in Equation 2 was calculated as: $A=floor\left(\left(scantime\right)/\left(labelduration+PLD+readoutduration\right)\right)$, where the floor function rounds down to the nearest integer. The noise variance used in the optimization and subsequent simulations was derived from preliminary in vivo data. All other model parameters are given in Table 1.

**Table 1:** Parameters used for optimizations, simulations, and in vivo experiments

Parameter	Value
General	
Label duration (τ)	1.4 s
*T*_1_ of blood (*T*_1*b*_)	1.65 s^34^
*T*_1_ of tissue (*T*_1*t*_)	1.445 s^35^
Labeling efficiency (α)	0.85^15^
Brain–blood water partition coefficient (λ)	0.9 mL/g^36^
Slice duration	53.125 ms
Slices (*N*)	5
Optimization	
Fixed CBF in apparent *T*_1_ (*T*_1_ʹ)	50 mL/100 g/min
Readout duration	1.275 s
In vivo experiments	
RF labeling pulse duration	600 µs (Gaussian)
RF labeling pulse separation	1 ms
RF labeling flip angle	20°
Mean labeling gradient	0.8 mT/m
Gradient during labeling pulses	6 mT/m
Nominal voxel size	3.4 × 3.4 × 5 mm
Matrix size	64 × 64
Partial Fourier factor	6/8
TE	21 ms
VENC	4 cm/s^4^

Abbreviations: CBF, cerebral blood flow; RF, radio‐frequency; TE, echo time; VENC, velocity encoding cutoff.

For this proof of principle study, the protocols were optimized so that the mean of Equation 9 across 5 slices was minimized. The number of slices was kept small to minimize the range of PLDs and BGS across slices. For a given slice, if all effective PLDs exceed the ATT then the FIM becomes severely ill‐conditioned because of a lack of ATT information. This is an inherent problem for CASL methods because the inflow of signal is more difficult to sample than for PASL. To avoid ill‐conditioned matrices, the ATT probability distribution was truncated for each slice based on the shortest attainable PLD. The sum in Equation 9 was then additionally weighted by the number of contributing slices. Analytical inversion of the FIM was performed to improve algorithm speed.

The optimized protocols were compared against a previously used evenly distributed multi‐PLD protocol^16^ (referred to as the reference multi‐PLD protocol) and a single‐PLD protocol using the recommended 1.8 s PLD.^4^ All protocols used a 1.4 s labeling duration to match the reference multi‐PLD protocol. The timings for these protocols are given in Tables 1 and 2. CRLB values were generated for each protocol across the ATT range. For the single‐PLD protocol, the CRLBs were estimated using $RMSE=\sqrt{{\mathrm{variance}}^{2}+{\mathrm{bias}}^{2}}$, where the bias is the systematic error caused by the assumed ATT (see below).

**Table 2:** Protocol timings

Protocol	Post‐labeling delays (s)	PLDs (*N*)	Averages (*N*)
Single‐PLD	1.8	1	33
Reference multi‐PLD	0.25, 0.5, 0.75, 1, 1.25, 1.5	6	7
CBF‐ATT_opt_	0.2, 0.2, 0.225, 0.3, 0.375, 0.45, 0.5, 0.55, 0.6, 0.6, 0.625, 0.625, 0.65, 0.65, 0.675, 0.675, 0.7, 0.7, 0.7, 0.7, 1.25, 1.275, 1.3, 1.35, 1.375, 1.4, 1.425, 1.425, 1.475, 1.5, 1.675, 1.75, 1.8, 1.825, 1.85, 1.875, 1.9, 1.925, 1.95, 1.975	40	1
CBF_opt_	0.2, 0.7, 0.825, 1, 1.125, 1.25, 1.325, 1.4, 1.475, 1.55, 1.625, 1.675, 1.7, 1.725, 1.75, 1.775, 1.8, 1.825, 1.85, 1.85, 1.875, 1.875, 1.9, 1.925, 1.925, 1.95, 1.975, 1.975, 2, 2.025, 2.025, 2.05, 2.075, 2.075	34	1

### 3.2 Simulation experiments

To validate the theoretical effects of the optimization, Monte Carlo simulations were performed for each ASL protocol. Two thousand sets of data were generated for each ATT value in the range 0.5≤ATT≤1.8 s, at 0.01 s intervals, using Equation 5 with the parameters in Table 1, for 5 slices. Gaussian white noise was added to label and control data before pairwise subtraction.

CBF and ATT were estimated from the data using Equation 5 and MATLAB’s non‐linear least squares (NLLS) function, “fmincon.” Fitting was initialized using a coarse grid search and bounded by 0≤CBF≤200 mL/100 g/min and 0≤ATT≤2.5 s. For the single‐PLD protocol, CBF was estimated by assuming ATT=1.25 s and fitting for CBF, because this resulted in the smallest theoretical error and uses the same assumptions as the multi‐PLD data. The RMSEs of the estimates, relative to the true values, were calculated for comparison. The RMSE represents a measure of accuracy and is a combination of both systematic bias and variance because of noise.

### 3.3 In vivo experiments

In vivo data was acquired for each ASL protocol to confirm that the theoretical benefits of the optimization were realized in practice. Seven healthy volunteers (3 female, 23–27 years old) were scanned under a technical development protocol, agreed with local ethics and institutional committees. All in vivo data was acquired on a 3T Prisma system (Siemens Healthcare, Erlangen, Germany) with a 32‐channel head coil. Additional scans performed were: a 3D multi‐slab time‐of‐flight angiography sequence, for placement of the PCASL‐labeling plane; a *T*_1_‐weighted structural image, for registration and tissue segmentation; and a *B*_0_ field map, for distortion correction of the ASL data.

For the PCASL data, imaging parameters were: single‐shot EPI readout; 5 transverse slices positioned to bisect the thalamus; nominal resolution = 3.4 × 3.4 × 5 mm^3^; matrix size = 64 × 64; bandwidth = 2004 Hz/pixel; TE = 21 ms; partial Fourier factor = 6/8, and fat saturation. The labeling plane was placed in a transverse orientation at the middle of the V3 section of the vertebral arteries, where the vertebral and internal carotid arteries are approximately parallel to each other and perpendicular to the transverse plane. PCASL labeling was achieved with: 600 µs duration Gaussian RF pulses, 1 ms spacing, 20° flip angle, and 1.4 s labeling duration. To reduce the impact of macrovascular signal, flow signal crushing was applied in the inferior–superior direction with a velocity encoding cutoff of 4 cm/s.^4^ BGS was achieved with a WET pre‐saturation module^22^ and 2 optimally timed global hyperbolic secant inversion pulses applied after PCASL labeling, as in previous studies.^16^, ^23^ Further ASL scan parameters are given in Table 1.

A fully relaxed *M*_0_ image was acquired with identical acquisition parameters to the PCASL data but without BGS and PCASL labeling. This was used as the reference volume for motion correction of the PCASL data and for voxel‐wise calibration.

To mitigate effects caused by changing physiology during the scan session, the PLDs from each of the ASL protocols were interleaved and distributed throughout the session. We noticed that large variations in the BGS effectiveness, such as when a short PLD followed a long PLD or vice versa, caused the online EPI *B*_0_ drift correction to create artificial sub‐voxel movements in the phase‐encode direction. To minimize this effect, the PLDs were not randomly ordered but were distributed across the scan session with the PLD duration gradually increasing and decreasing in 4 cycles. This resulted in a well‐distributed coverage across the total ASL scan duration for each protocol but also maintained gradual variations in the PLDs and resulting BGS performance therefore minimizing erroneous shifting in the image. Four extra PLDs were also acquired to ensure a maximum spacing of 75 ms across the acquired PLD range (see Table 2). This was used for the ground truth data (described below) to ensure a high temporal sampling of the data without any significant gaps.

### 3.4 Post‐processing

GM masks were generated from the *T*_1_ structural image using FSL’s FAST^24^ tool. The ASL images were motion corrected using FSL’s FLIRT^25^ with 3 degrees of freedom. Distortion correction was performed using the *B*_0_ field map and FSL’s FUGUE tool. Further registrations were performed to transform the GM mask into the ASL native space, thresholding it at 50%. Before voxelwise calibration, the *M*_0_ image was smoothed with a Gaussian kernel (σ=2.5 mm), as recommended in the recent consensus paper.^4^

The kinetic model was fit to the in vivo data exactly as described for the simulated data above. To evaluate the error associated with the CBF and ATT estimates from each protocol, ground truth estimates were generated by combining and fitting all un‐averaged ASL data across all the protocols, so as to equally weight the data from each protocol. The CBF and ATT errors from each protocol were then calculated relative to these ground truth estimates. To ensure only well‐fit ground truth GM estimates were used in further analysis, the following restrictions were imposed. The CRLB (Equations (1) and (2)) was calculated voxel‐wise and used as an approximation of the ground truth CBF and ATT variance (uncertainty). The noise was estimated by the summed squared residuals of each fit, normalized by the statistical degrees of freedom.^26^ Voxels were excluded if the estimated ground truth CBF and ATT SD were >5 mL/100 g/min and 0.1 s, respectively. The analysis was further restricted to voxels with ground truth ATT estimates in the range of interest, 0.5≤ATT≤1.8 s. The RMSE of the estimates from each protocol, relative to the ground truth values, were calculated for comparison. A 2‐tailed non‐parametric test (Wilcoxon signed rank test) was used to test for significant differences across subjects (*P* < 0.05).

## 4 RESULTS

### 4.1 PLD optimization

**Figure 3:**
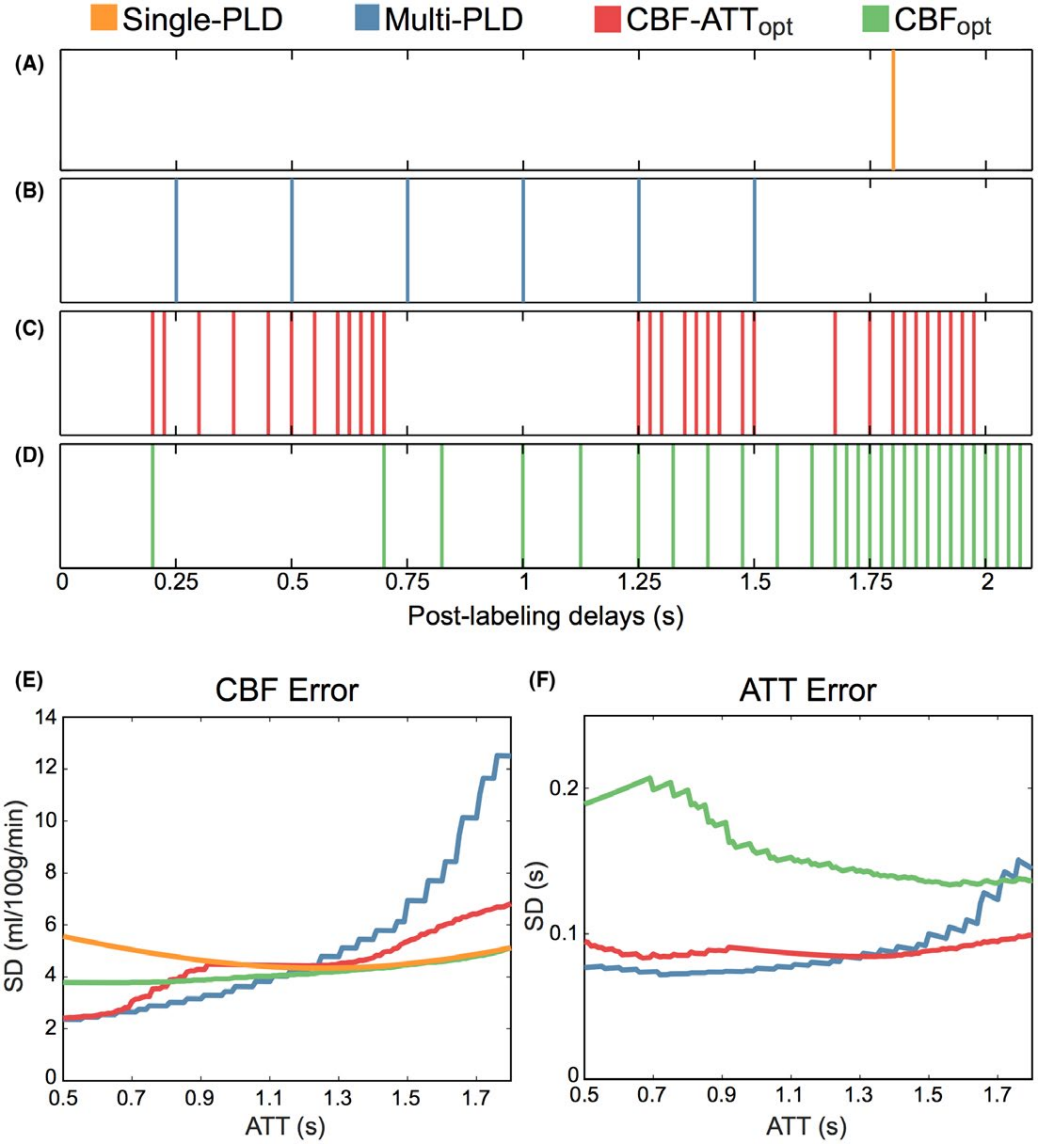
The PLDs (A–D) and the predicted CBF and ATT errors (Cramér‐Rao lower bound [CRLB] SD) (E and F) for each of the protocols. The reference single‐PLD protocol (A) has a fixed PLD at 1.8 s, the reference multi‐PLD protocol (B) uses evenly distributed PLDs between 0.25–1.5 s, whereas the optimized protocols, CBF‐ATT_opt_ (C) and CBF_opt_ (D), have more targeted PLDs. Repeated PLDs are not shown, but are listed in full in Table 2. The CRLB SDs for CBF and ATT demonstrate the impact that the choice of PLDs have on inference accuracy

The PLDs for each protocol are given in Table 2 and shown in Figure 3. Both optimized schemes have very distinct sampling patterns with a high number of PLDs with few repeats. The CBF‐ATT_opt_ protocol has 2 main groups: a group of short PLDs, sampling signal inflow, and a group of longer PLDs, sampling the signal peak and decay for most of the ATT distribution. The CBF_opt_ protocol covers a similar range of PLDs, but has very few short PLDs and increased density of longer PLDs, leading to more sampling of the signal curve after complete bolus arrival.

The theoretical CBF and ATT errors (CRLBs) are also shown in Figure 3 and demonstrate the predicted improvement in accuracy of the optimized protocols across the ATT prior range. Both optimized protocols have less variable CRLB profiles than the reference multi‐PLD protocol, whereas the CBF_opt_ protocol demonstrates the lowest average CBF error. The increase in the ATT CRLB of the CBF_opt_ protocol is not reflected in its CBF errors because of its designed insensitivity to ATT accuracy. The single‐PLD protocol achieves its minimal error at ATT=1.25 s but is less accurate than the CBF_opt_ protocol.

### 4.2 In vivo data selection

**Figure 4:**
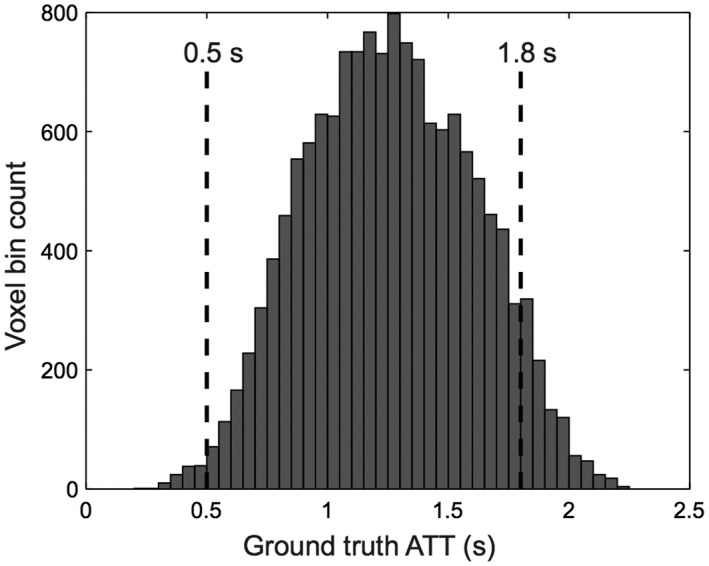
Histogram of the ground truth ATT estimates that had an estimated CBF and ATT maximum likelihood distribution SD <5 mL/100 g/min and 0.1 s, respectively. The range of ATTs included in further analysis are shown by vertical dashed lines

Before presenting the MC simulation and in vivo results together, we briefly describe the results of the in vivo data selection. Across the 7 subjects, the GM masks contained 19,732 voxels in total. Of these, 31.6% were excluded because they did not meet one or more of the restrictions imposed on the ground truth data: 26.3% did not meet the uncertainty restrictions and a further 5.3% were not in the specified ATT range. A histogram of the ATTs for the well‐fit ground truth voxels is shown in Figure 4, demonstrating that the majority of well‐fit voxels were within the targeted ATT range (94.7%). ATTs outside the range 0.5≤ATT≤1.8 s were not used in further analysis.

### 4.3 Protocol comparison

**Figure 5:**
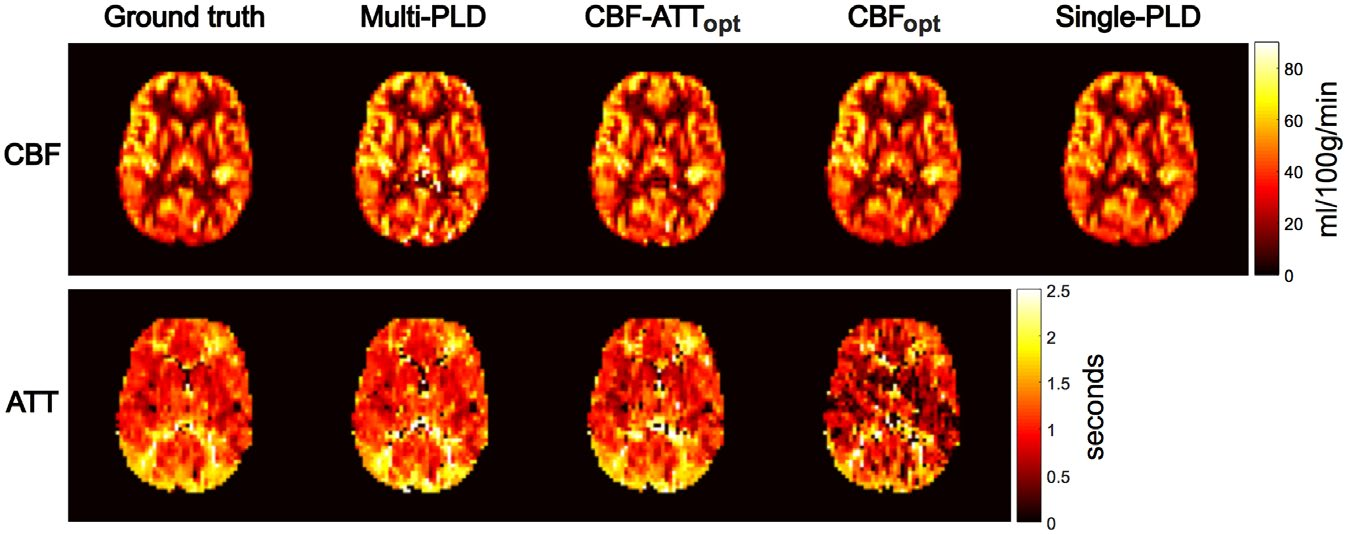
Representative CBF (top) and ATT (bottom) maps for the ground truth estimates and the 4 tested protocols. The maps show an axial slice from a single subject. Note there is no ATT map for the single‐PLD protocol

Representative CBF and ATT maps for 1 subject are shown in Figure 5. There is a reasonable qualitative agreement between the CBF maps. However, the reference multi‐PLD protocol map exhibits noticeably more noise. Good agreement between the ATT maps for ground truth, reference multi‐PLD, and CBF‐ATT_opt_ protocols can be seen, whereas the CBF_opt_ ATT map is clearly less accurate.

**Figure 6:**
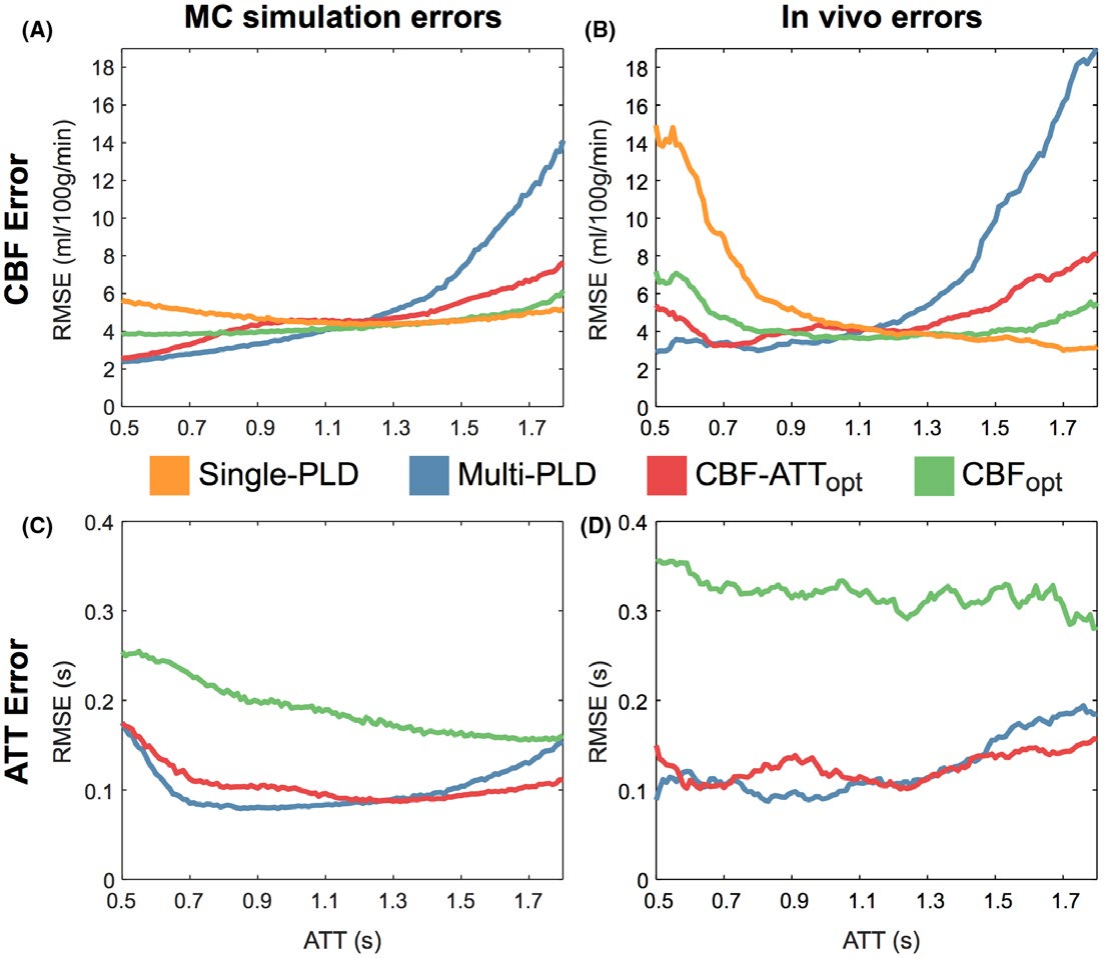
CBF (top) and ATT (bottom) RMSEs for the Monte Carlo simulations (A and C) and in vivo experiments (B and D). The in vivo data are the combined data across all 7 subjects, which has been smoothed using a sliding window mean (window width = 100 ms; increment = 10 ms)

Figure 6 shows the RMSE in CBF and ATT estimation for each protocol from the MC simulations and the in vivo data across the ATT range. For the in vivo data, all 7 subjects’ data were combined using a sliding window to aid interpretability. There is a strong correspondence between the MC simulations and in vivo data, which both agree extremely well with the trends seen in the predicted errors (Figure 3), demonstrating the expected improvement in CBF and ATT estimation with the optimized protocols. As expected, the CBF_opt_ protocol resulted in high CBF accuracy while having less accurate ATT estimates.

**Figure 7:**
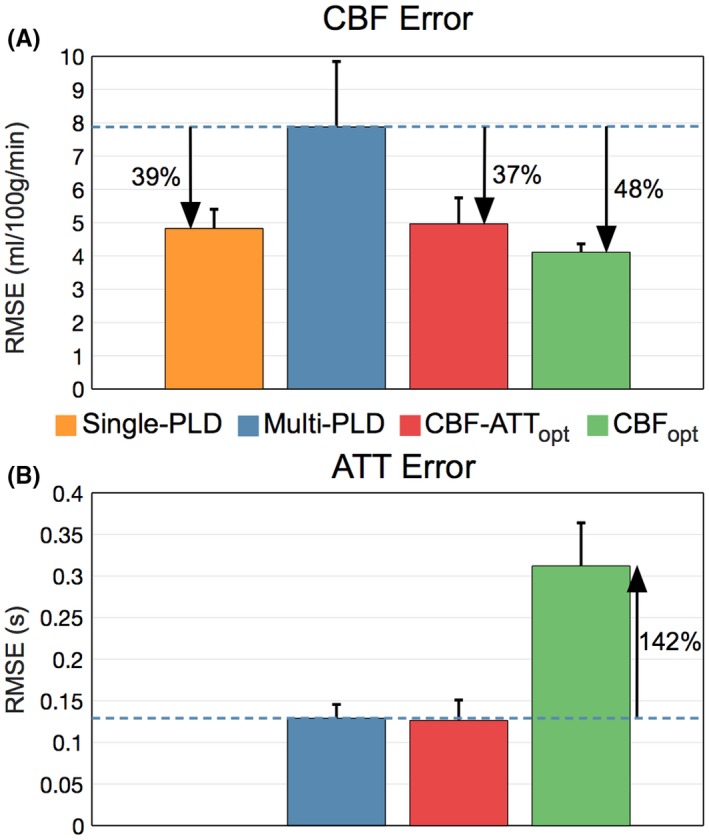
In vivo CBF (A) and ATT (B) RMSEs across subjects. The height of each bar graph is the mean RMSE across subjects, while the error bar shows the SD across subjects. The errors were checked for significant differences using a non‐parametric paired test (Wilcoxon signed rank test), *P* < 0.05. All differences are significant, except between the single‐PLD and CBF‐ATT_opt_ CBF errors and reference multi‐PLD and CBF‐ATT_opt_ ATT errors

The average in vivo RMSEs across subjects are shown in Figure 7. The CBF RMSEs were (mean ± SD mL/100 g/min): 4.11 ± 0.25 (CBF_opt_), 4.83 ± 0.57 (single‐PLD), 4.97 ± 0.78 (CBF‐ATT_opt_), and 7.88 ± 1.97 (reference multi‐PLD). Note that both optimal protocols had significantly reduced CBF errors compared to the reference multi‐PLD protocol, whereas CBF_opt_ also had significantly reduced CBF errors compared to the single‐PLD protocol. The CBF_opt_ ATT RMSE (0.31 ± 0.05 s) was significantly higher than the reference multi‐PLD (0.13 ± 0.02 s) and CBF‐ATT_opt_ (0.13 ± 0.02 s) ATT RMSE. These results are very consistent across subjects, as shown in Supporting Information Figure S2, demonstrating their robustness.

**Figure 8:**
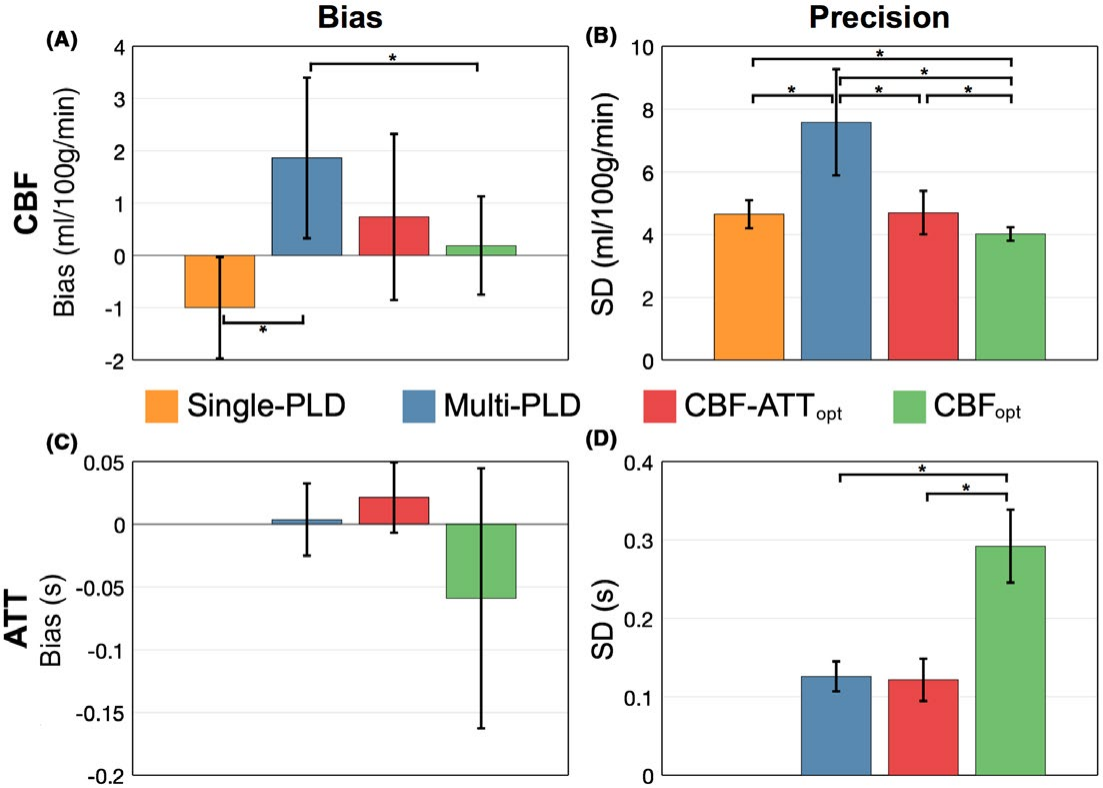
In vivo CBF (top) and ATT (bottom) bias (A and C) and precision (B and D) across subjects. The height of each bar graph is the mean across subjects, whereas the error bar shows the SD across subjects. An asterisk (*) signifies significant differences using a non‐parametric paired test (Wilcoxon signed rank test), *P* < 0.05

The RMSE is a useful metric for comparison because it combines both the bias and variance in the estimates. However, we can also separately examine these measures. This breakdown is shown for the in vivo data in Figure 8. For the CBF estimates, the CBF_opt_ protocol had a significantly smaller bias and SD than both the reference single‐PLD and multi‐PLD protocols, while the CBF‐ATT_opt_ protocol had a significantly lower SD than the reference multi‐PLD protocol but not the single‐PLD protocol. For the ATT estimates, there were no significant differences in the biases (although a trend of underestimation can be seen for CBF_opt_) whereas CBF_opt_ had significantly higher SD than the reference multi‐PLD and CBF‐ATT_opt_ protocols.

## 5 DISCUSSION

In this work, we have presented a general framework for optimizing ASL experiments. This framework can be used with any labeling scheme, for which there is an analytical model, and any readout. We demonstrated the practical benefits of using this framework in the specific case of fixed label duration, multi‐PLD PCASL experiments with a 5‐slice EPI readout. We designed 2 protocols that either aimed to improve both CBF and ATT accuracy or just CBF accuracy. These protocols were shown to achieve their respective aims using Monte Carlo simulations and in vivo experiments, including strong agreement with the predicted performance. The CBF optimized protocol resulted in a 48% reduction of in vivo CBF errors relative to the reference multi‐PLD protocol, while optimizing for both CBF and ATT resulted in a 37% reduction of in vivo CBF errors, without a loss in ATT accuracy. We have also shown that a well‐optimized multi‐PLD protocol can produce more accurate CBF estimates (15% reduction in error) than a single‐PLD protocol in the same scan time, as well as providing potentially physiologically useful ATT maps.^6^

### 5.1 Optimization

Simplifications that we have made to the FIM (see Theory and Supporting Information Text S2) show that a CBF distribution is not required for optimization because the optimal protocol is insensitive to the value chosen. These simplifications improve usability compared to previous PASL work,^11^, ^12^, ^13^ because only an ATT prior probability distribution needs to be defined, and they also greatly speed up the optimization because of the reduced dimensionality of the problem. Furthermore, by including the number of averages in Equation (2), the scan time can also be pre‐specified, meaning that strict clinical scan times can be adhered to and the optimal protocol for the time available may be found.

Equation 9, as used in the algorithm outlined in Figure 2, combines the CRLB information across the ATT distribution before finding the optimal timing for each PLD. This approach results in PLD timings that maximize the mean information obtained across the entire ATT prior probability distribution. This is a distinct advantage over previous work where the PASL inversion times (TIs) were chosen based on a histogram of locally optimal TIs for individual ATT and CBF parameter pairs drawn from the priors, which was divided between a fixed number of TIs.^11^, ^12^ Further PASL work used an average approach, using an adaptive quadrature technique to reduce the number of samples required from the ATT and CBF priors therefore improving the speed of optimization.^13^ However, after removing the need for a CBF prior distribution, the number of calculations required is drastically reduced, limiting the benefit of using such a technique. The direct sampling method we have used is also more easily implemented, assisting the adoption of CRLB optimization for ASL studies.

We have proposed the use of a uniform ATT prior probability distribution, rather than a normal distribution.^11^, ^12^, ^13^ ATT has been shown to be regionally dependent,^27^ therefore even if a pre‐defined normal distribution does exactly match the distribution of ATTs found in a given subject, the brain regions corresponding to the edges of the distribution will be less accurately estimated than regions corresponding to the center of the distribution. This may be desirable if certain brain regions are of more interest to the experimenter, but we have opted to equally weight the entire brain in this study. However, the general framework described here would allow any shape or range of ATT distribution to be used.

The ATT range used in this work was appropriate for healthy volunteers.^4^, ^20^ This is the main a priori knowledge necessary for optimization using this method but may not always be known. When scanning specific patient groups where the typical ATT range is known, this should be used to inform the ATT prior probability distribution. However, when no specific a priori information is available, a conservative large range of ATTs could be used.

We found the algorithm to be reasonably insensitive to initialization, with PLDs in the final protocol only varying by 1 or 2 increments of 25 ms if different initial conditions are chosen. This suggests that solutions close to the global minimum were achieved. It should be noted that care must be taken to initialize the PLDs to cover most of the ATT range. If not, the PLDs may fail to update because of all possible PLD choices being dominated by ill‐conditioned FIMs within the ATT distribution.

### 5.2 Optimal protocols

The optimized multi‐PLD protocols found in this work differ greatly from commonly used evenly spaced multi‐PLD protocols. The designs reflect the underlying information content, demonstrated by the sensitivity functions (Figure 1). For a single ATT value, the CBF‐ATT_opt_ PLDs were found at PLD=[ATT,ATT+τ], as found by Xie et al.^11^ However, the CBF‐ATT_opt_ protocol did not simply reflect this relationship and the shape of the ATT prior. Instead, a starkly different pattern is clearly seen in Figure 3, which is a result of the optimization accounting for the combined information obtained across all PLDs simultaneously, rather than considering the local optimization of each PLD.

We also introduced the CBF_opt_ protocol, which minimizes the CBF variance, while being insensitive to ATT variation. Xie et al.^11^ also used a CBF‐specific optimization, but did not include the ATT sensitivity function in the FIM. If the ATT sensitivity function is not included in the FIM, it is implicitly assumed that the ATT is known, which results in much larger errors (see Supporting Information Figure S3).

An alternative optimality criterion not investigated in this work is the non‐zero weighted trace of the CRLB matrix in Equation (4). This could be used to choose any trade‐off between CBF and ATT accuracy, resulting in a much more flexible tool for optimizing ASL experiments. However, the relative weightings for the desired trade‐off would need to be empirically deduced; a potentially burdensome process. Furthermore, other model parameters, such as tissue T_1_, could be included in the FIM with the aim of minimizing sensitivity to them, similar to the CBF_opt_ approach to ATT.

For both protocols, the optimal number of PLDs were found to be large because a large number of PLDs provides the most flexibility for minimizing the predicted variance. This may seem counterintuitive, because each data point is relatively noisy. However, the information across PLDs is combined during fitting, giving similar results to averaging, but with greater information content. For ease of implementation, the proposed framework can be restricted to choose a smaller number of PLDs with more than 1 average at each. For example, restricting the number of CBF‐ATT_opt_ PLDs to 10 or fewer resulted in a 10 PLD protocol which only increased the predicted mean CBF and ATT errors by ~0.5% and ~1%, respectively (see Supporting Information Figure S4). This relatively small increase suggests that a smaller number of PLDs can still be used effectively.

In this study, the PCASL labeling duration was fixed at that used in the reference protocol^16^ for simplicity. However, using this framework, a single label duration for the whole experiment or different values for each individual PLD could be optimized, yielding greater flexibility in the design and potentially leading to further reductions in CBF and ATT estimation errors. Such extensions to this approach will be explored in future work. Furthermore, the sub‐boli timings in time‐encoded PCASL,^28^ a method that efficiently produces multi‐delay ASL data, could be optimized, potentially yielding greater improvements in estimation accuracy. We are currently investigating the potential of these more advanced techniques.

### 5.3 Simulation and in vivo experiments

Figures 3 and 6 demonstrate the excellent agreement between the theoretical, simulated, and in vivo CBF and ATT errors, validating the optimization framework outlined here. However, there was a poorer agreement between the simulation and in vivo CBF results at ATTs <0.8 s, where the CBF errors for all protocols were higher than expected, with the single‐PLD protocol being most affected. This could be because of the reduced data available at these ATT values, making the results more susceptible to noisy estimates, or the presence of residual macrovascular signal causing CBF overestimates in the ground truth data. Another noticeable difference was the increase in the simulation ATT RMSE at short transit times, compared to the CRLBs. This is because of poor ATT estimation in the top few slices that was not possible to resolve because of the longer effective PLDs at these slices.

The CRLB is based on maximum‐likelihood estimation, as is NLLS fitting. However, the data is not restricted to being fit with a NLLS method, though we chose it here for consistency. Supporting Information Figure S5 demonstrates the differences between fitting the in vivo data with the NLLS approach and a variational Bayesian approach, as implemented in the BASIL toolbox in FSL,^29^ which is commonly applied to in vivo data. The general trends of the protocols are very similar for both fitting methods, and therefore this choice does not influence the conclusions of this article.

Although the majority of well‐fit voxels in the healthy volunteers were within the ATT range optimized for, 4.8% had ATTs above 1.8 s. If the optimization ATT range was extended, the number of well‐fit voxels with longer ATTs would probably increase. For this reason, future work in healthy volunteers should consider using a wider ATT prior.

To demonstrate the robustness of the results to the ground truth data exclusion criteria, we re‐analyzed all of the GM data without the restrictions on fitting accuracy or ATTs placed on the ground truth data (see Supporting Information Figure S6). The trends and relative performance seen in Figures 6 and 7 are largely unchanged, although with larger RMSE for all protocols.

The in vivo CBF and ATT estimates were compared with ground truth estimates, derived from the same, but combined, data from the individual protocols. This has the potential to benefit the accuracy of some protocols over others if the estimates are biased in some way. However, when we simulated this analysis process, the relative performance of each protocol was largely unaffected. Specifically, using estimated ground truth values lead to a relatively even decrease in the CBF and ATT RMSE across all of the protocols: 0.49 ± 0.08 mL/100 g/min and 0.011 ± 0.004 s, respectively.

Magnitude data has a Rician noise distribution, though we assumed Gaussian distributed noise in our simulations and data fitting. It has been shown, however, that when the SNR of data is >3, a Gaussian distribution is a good approximation of the true Rician distribution.^30^ To test if this condition was met with the in vivo ASL data used in this study, we calculated the temporal SNR (tSNR) of the reference multi‐PLD and single‐PLD control GM magnitude data using the formula: tSNR = S/σ, where S and σ are the mean and SD of the signal over the repeats for each PLD. Only 5.4% of the GM voxels had a tSNR <3 with the median tSNR being 31.4. However, if extremely efficient BGS was used or complex subtraction performed before magnitude reconstruction of the perfusion weighted data, then a Gaussian distribution would no longer be an appropriate approximation.

In the optimization and fitting, we assumed an identical noise magnitude across PLDs. However, the BGS strategy we used in this work resulted in variable static tissue signal suppression and therefore variable noise levels across the PLDs (see Supporting Information Figure S7). Using the measured noise amplitude across PLDs in MC simulations resulted in minor differences to the CBF and ATT errors across the different protocols, but the broad trends were unchanged (see Supporting Information Text S3 and Supporting Information Figure S8). The variable noise could be incorporated into the optimization, but it is highly specific to the sequence used. Its effect on the estimates could also be reduced by weighting the data appropriately during fitting.^29^ Alternatively, a more flexible BGS scheme that allows interleaving of the inversion pulses with labelling, such as that used by Dai et al.,^8^ would result in more comparable BGS across PLDs, better meeting our assumption of equal noise at all PLDs. A more even level of BGS across PLDs will also further reduce the small image shift artefacts we noticed when the BGS varied.

For this proof‐of‐concept study, we used a multi‐slice 2D readout with a small number of slices to minimize the variation in timings and BGS. However, the use of a 3D readout would ensure identical BGS and PLDs across all slices, leading to a simpler optimization problem and improved static tissue attenuation across the imaged volume. This would enable the full benefits of this optimization framework to be realized for whole brain perfusion measurements. Although we only validated this framework in a limited cross‐section of the brain, we expect this method to benefit perfusion measurements across the whole brain because the range of ATTs in our data (Figure 4) are comparable to previously reported ranges across the whole brain,^8^, ^20^, ^31^ and inferior–superior ATT variations have previously been shown to be comparable to within‐slice variations.^23^

Because of the increasing use of 3D readouts with ASL,^4^ we have included 3D (or single‐slice) specific CBF‐ATT_opt_ and CBF_opt_ PLD timings for completeness. These were generated for 2 ATT ranges: a standard ATT range of 0.5 ≤ ATT ≤ 2 s and a prolonged ATT range of 1 ≤ ATT ≤ 3 s, which were compared to an evenly distributed protocol in each case. The timings can be found in Supporting Information Table S1 and described in Supporting Information Text S4. In each case, the number of PLDs has been constrained to ≤10 to facilitate acquisition segmentation, necessary to reduce blurring or distortions. The PLD timings and MC simulation RMSEs are shown in Supporting Information Figure S9. For both ATT ranges, the optimized protocols reduced the average errors across the ATT distributions relative to the evenly distributed protocol. However, for late ATTs in the prolonged ATT range, the CBF and ATT errors were large for all protocols, demonstrating the difficulty in estimating CBF in the presence of delayed ATT and short scan durations. Future work will explore improved methods for CBF and ATT estimation in delayed ATT cases, including optimizing the label durations and the use of time‐encoded ASL. An alternative method that holds promise for CBF estimation in the presence of delayed ATT is velocity‐selective ASL,^32^ which effectively eliminates the ATT and so experiences minimal tracer T_1_ decay compared to whole brain PCASL.

We have focused on optimizing protocols for GM in this study. However, the framework could also be applied to measuring white matter (WM) perfusion, which typically has longer ATTs, shorter T_1_ and lower CBF than GM, making it a much more challenging application, increasing the importance of appropriately optimizing protocols. This interesting application will be investigated in future work. The low SNR of WM could also be aided by moving to higher field strengths than 3T because of longer T_1_ relaxation times.^33^

It should be noted that the reference multi‐PLD protocol was originally intended for a 24‐slice, whole brain scan, meaning that the average effective PLDs across the brain are on the order of 0.5 s longer than those given in Table 2. This would likely result in more accurate CBF estimates at longer ATTs in higher slices than is presented here.

## 6 CONCLUSIONS

In conclusion, we have developed a general framework for optimizing ASL experiments and validated this approach in the specific case of multi‐PLD PCASL, showing significant improvements over reference single‐PLD and multi‐PLD protocols. The clinical use of this framework will lie in the development and testing of standardized, patient population‐specific protocols.

## CONFLICT OF INTEREST

Michael Chappell receives royalties from commercial licensing of the FMRIB software library.

## Supporting information


**FIGURE S1** The effect of true CBF on estimation errors in CBF (top) and ATT (bottom). Both the predicted Cramér‐Rao lower bound (A and C) and RMSEs for Monte Carlo simulations (B and D) are shown. These results demonstrate that the CBF estimation errors do not vary greatly with CBF, whereas the ATT errors are inversely proportional to CBF. The reference multi‐PLD protocol for 1 slice was used for this demonstration
**FIGURE S** **2** In vivo CBF (A) and ATT (B) RMSEs for each of the 7 subjects. The trends are extremely similar across the subjects, demonstrating the robustness of the optimization
**FIGURE S3** The PLDs (A and B) and the predicted CBF and ATT errors (Cramér‐Rao lower bound [CRLB] SD) (C and D) for CBF_opt_ with and without the ATT sensitivity function in the FIM. We assumed a 0.5‐s readout duration for a 3D acquisition, 28 PLDs, and an ATT range of 0.5–2 s (similar to Supporting Information Text S4). Repeated PLDs are not shown. CBF and ATT CRLBs at ATTs shorter than 0.75 s are extremely large for the protocol without the ATT sensitivity function included and are out of view for clarity. The differences in the chosen PLDs and the resulting CRLBs demonstrate the importance of including the ATT sensitivity function in the FIM. If the ATT sensitivity function is not included in the FIM, then it is implicitly assumed that the ATT is known, which can result in large errors
**FIGURE S4** The PLDs (A and B) and the predicted CBF and ATT errors (Cramér‐Rao lower bound [CRLB] SD) (C and D) for the CBF‐ATT_opt_ protocol with 10 or 40 PLDs. The 10 PLD protocol uses 4 averages of the 10 PLDs, whereas the 40 PLD protocol only has 1 average for each PLD. Using 10 PLDs rather than 40 PLDs only resulted in a ~0.5% and ~1% increase in the CBF and ATT CRLBs, respectively
**FIGURE S5** In vivo RMSEs of CBF (top) and ATT (bottom) for data fitted with the NLLS method, as in Figure 6 (A and C), and with BASIL^29^ (B and D), a variational Bayesian algorithm. The fitting priors used for the BASIL fitting were (mean ± SD): 0 ± 106 mL/100 g/min and 1.25 ± 1 s, for CBF and ATT, respectively. Similarly to the NLLS data, only BASIL fitted data that had CBF and ATT posterior distribution SD <5 mL/100 g/min and 0.1 s, respectively, were included in the graph. BASIL reduced CBF errors, particularly in regions with very large errors in the NLLS fitting. Mean ATT errors were also reduced, but were larger at short ATTs. This suggests that BASIL produces better CBF estimates from noisy data than a naive NLLS algorithm, but there remain significant benefits from appropriately optimizing the PLDs in both cases
**FIGURE S6** In vivo RMSEs of CBF (left column) and ATT (right column) estimates for all GM data for the 7 subjects with no other data exclusion criteria imposed. The top row (A and B) shows the RMSE trends across ATTs, whereas the bottom row (C and D) shows the mean and SD of the RMSEs across subjects. The general trends remain unchanged, compared to Figures 6 and 7. As expected, there is a general increase in RMSEs compared to Figures 6 and 7 because of the removal of the ground truth data exclusion criteria. Graphs (A) and (B) agree well with Figure 6 in the ATT prior range of 0.5 ≤ ATT ≤ 1.8. The trends in (C) and (D) also agree well with Figure 7, although the CBF_opt_ and single‐PLD CBF RMSEs are no longer significantly different. This is to be expected because there is greater noise in the ground truth estimates and data from outside the optimized range has been included
**FIGURE S7** Boxplots of the tSNR, signal, and noise of the GM voxels from the control ASL data of the reference multi‐PLD and single‐PLD data for all 7 subjects. The median tSNR has been fit using an exponential decay model, whereas the tissue signal (normalized by *M*_0B_) was simulated using a series of saturation and inversion recovery models. The noise can then be modeled as the simulated signal divided by the fitted tSNR
**FIGURE S8** The effect on CBF (A and B) and ATT (C and D) RMSEs when using uniform (A and C) or variable (B and D) noise across PLDs. Details of the simulations are given in Supporting Information Text S3. The broad trends are consistent. However, all protocols except the reference multi‐PLD protocol have reduced CBF errors at late ATTs and the ATT errors for all protocols increased across the entire ATT range. The single‐PLD, CBF‐ATT_opt_, and CBF_opt_ CBF estimates benefit from having many PLDs at times of reduced noise with the variable noise model
**FIGURE S9** The PLDs (A and D) and the MC simulation RMSEs (B, C, E, and F) for Even, CBF‐ATT_opt_, and CBF_opt_ protocols over a standard healthy range of ATTs (0.5 ≤ ATT ≤ 2 s) and a prolonged ATT range (1 ≤ ATT ≤ 3). The timings shown are for a 3D acquisition. Repeated PLDs are not shown, but are listed in full in Supporting Information Table S1
**TABLE S1** Optimal PLDs for 3D acquisitions
**TEXT S1** Complete CBF sensitivity function
**TEXT S2** Optimal design independence to CBF
**TEXT S3** Variable noise Monte Carlo experiments
**TEXT S4** PLD optimization for 3D acquisitions

## APPENDIX 1

The fitted data and analysis code that underpin the data and figures in this work can be accessed via the Zenodo repository (https://doi.org/10.5281/zenodo.1291502).
