# Supplementary material for: A general framework for optimizing arterial spin labeling MRI experiments
Source: Magn Reson Med. 2018 Dec 26;81(4):2474–88. doi: 10.1002/mrm.27580 (PMC6492260; doi:10.1002/mrm.27580)
Supplement: Supplementary file 1 — FIGURE S1 The effect of true CBF on estimation errors in CBF (top) and ATT (bottom). Both the predicted Cramér‐Rao lower bound (A and C) and RMSEs for Monte Carlo simulations (B and D) are shown. These results demonstrate that the CBF estimation errors do not vary greatly with CBF, whereas the ATT errors are inversely proportional to CBF. The reference multi‐PLD protocol for 1 slice was used for this demonstration FIGURE S 2 In vivo CBF (A) and ATT (B) RMSEs for each of the 7 subjects. The trends are extremely similar across the subjects, demonstrating the robustness of the optimization FIGURE S3 The PLDs (A and B) and the predicted CBF and ATT errors (Cramér‐Rao lower bound [CRLB] SD) (C and D) for CBFopt with and without the ATT sensitivity function in the FIM. We assumed a 0.5‐s readout duration for a 3D acquisition, 28 PLDs, and an ATT range of 0.5–2 s (similar to Supporting Information Text S4). Repeated PLDs are not shown. CBF and ATT CRLBs at ATTs shorter than 0.75 s are extremely large for the protocol without the ATT sensitivity function included and are out of view for clarity. The differences in the chosen PLDs and the resulting CRLBs demonstrate the importance of including the ATT sensitivity function in the FIM. If the ATT sensitivity function is not included in the FIM, then it is implicitly assumed that the ATT is known, which can result in large errors FIGURE S4 The PLDs (A and B) and the predicted CBF and ATT errors (Cramér‐Rao lower bound [CRLB] SD) (C and D) for the CBF‐ATTopt protocol with 10 or 40 PLDs. The 10 PLD protocol uses 4 averages of the 10 PLDs, whereas the 40 PLD protocol only has 1 average for each PLD. Using 10 PLDs rather than 40 PLDs only resulted in a ~0.5% and ~1% increase in the CBF and ATT CRLBs, respectively FIGURE S5 In vivo RMSEs of CBF (top) and ATT (bottom) for data fitted with the NLLS method, as in Figure 6 (A and C), and with BASIL29 (B and D), a variational Bayesian algorithm. The fitting priors used for the BASIL fitt [file MRM-81-2474-s001.pdf]

Supporting Information Text S1: Complete CBF sensitivity function

The CBF sensitivity function with variable CBF in the apparent T_1 of tissue (T_1') is:

$$\frac{\partial \Delta M(t)}{\partial f} = 0 \qquad 0 < t < \Delta t$$

$$= 2M_{0B}\alpha e^{\frac{-\Delta t}{T_{1b}}}\left[T_1'\left(1 - e^{\frac{-(t-\Delta t)}{T_1'}}\right) + \left(\frac{fT_1'(t-\Delta t)}{\lambda} - f\frac{\partial T_1'}{\partial f}\right)e^{\frac{-(t-\Delta t)}{T_1'}} + f\frac{\partial T_1'}{\partial f}\right] \qquad \Delta t < t < \tau + \Delta t \tag{10}$$

$$= 2M_{0B}\alpha e^{\frac{-\Delta t}{T_{1b}}}\left[T_1' e^{\frac{-(t-\tau-\Delta t)}{T_1'}}\left(1 - e^{\frac{-\tau}{T_1'}}\right) + \left(\frac{fT_1'(t-\Delta t)}{\lambda} - f\frac{\partial T_1'}{\partial f}\right)e^{\frac{-(t-\Delta t)}{T_1'}} + \left(f\frac{\partial T_1'}{\partial f} - \frac{fT_1'(t-\tau-\Delta t)}{\lambda}\right)e^{\frac{-(t-\tau-\Delta t)}{T_1'}}\right] \qquad \tau + \Delta t < t$$

where $\frac{\partial T_1'}{\partial f} = -\frac{\lambda T_{1t}^2}{(\lambda + fT_{1t})^2}$. The ATT sensitivity function is the same as Equation 7.

Supporting Information Text S2: Optimal design independence to CBF

By assuming a fixed value of CBF in the apparent T_1 of tissue (T_1'), we have that the CBF sensitivity function is independent of CBF, while the ATT sensitivity is linearly proportional to CBF:

$$\frac{\partial \varDelta M(t)}{\partial \varDelta t} \propto f \tag{11}$$

It then follows that the CRLB CBF variance (also the L-optimality criterion) is independent from the CBF while the CRLB ATT variance is inversely proportional to the square of the CBF:

$$\sigma_{\varDelta t}^2 \propto \frac{1}{f^2} \tag{12}$$

It also follows that the D-optimality criterion (the determinant of the CRLB) is inversely proportional to f^2:

$$\phi(t;\theta)_{D-optimal} = \frac{1}{det(F(t;\theta))} \propto \frac{1}{f^2} \tag{13}$$

Supporting Information Figure S1 demonstrates, using MC simulations, that the CBF RMSE does not vary with CBF, while the ATT RMSE decreases globally with increasing CBF, as predicted by the CRLB SD.

Since both cost functions used in this work either scale with or have no relationship to CBF, a design which is optimal for one value of CBF will be optimal for another. This is also approximately true for the full sensitivity functions case.

<u>Supporting Information Text S3</u>: Variable noise Monte Carlo experiments

It is clear from *Supporting Information Figure S7* that the noise magnitude is not constant across PLDs. We used the reference multi-PLD and single-PLD control data here because multiple averages at each PLD can be used to calculate the average signal and SD voxel-wise at each PLD. The noise magnitude appears to vary in a similar manner to the residual tissue signal, decreasing as the PLD approaches 1.25 s then increasing for longer PLDs. We ran Monte Carlo simulations including a model of the variable noise magnitude in order to measure the effect that variable noise can have on the CBF and ATT estimates. Since the noise varies in a non-simple manner, we simulated the residual tissue signal and fit a model to the tSNR across PLDs. We can then simply divide the residual tissue signal by the tSNR at any PLD to get an estimate of the PLD dependent noise.

We simulated the residual tissue signal after BGS using a series of simple saturation and inversion recovery calculations using the scanner inversion timings and assuming perfect presaturation and inversion pulses. We fit the median tSNR with an exponential decay model of the form $tSNR(t) = a \cdot e^{-b \cdot t} + c$, where t is the label duration plus the PLD and $a,\ b$ and c are constants to be estimated. The noise SD can then be estimated as $\sigma(t) = signal(t)/tSNR(t)$.

The results of the residual signal simulations, tSNR fitting and noise estimation are shown overlaid on the in vivo data for all 7 subjects in *Supporting Information Figure S7*. The noise SD was scaled such that the average SD was identical to that used in the previous simulations. We performed and analyzed the Monte Carlo simulations in an identical manner to the simulations performed for *Figure 6*, which are described in the methods section of the article, except that variable noise SD was used instead. The RMSEs of the CBF and ATT estimates for each protocol are shown in *Supporting Information Figure S8* alongside the RMSEs of the uniform noise estimates. While the reference multi-PLD protocol CBF RMSE increased by an average of 0.80 ml/100g/min across the ATT range, the other protocols decreased by 0.62 ml/100g/min (CBF-ATT_{opt}), 1.02 ml/100g/min (CBF_{opt}), and 1.47 ml/100g/min (single-PLD), reflecting the reduced noise due to the PLD placement of each protocol. All of the protocols had increased ATT RMSE, by: 0.074 s (reference multi-PLD), 0.088 s (CBF-ATT_{opt}), and 0.162 s (CBF_{opt}), presumably because of the increased uncertainty due to equally weighting the data despite there being varying noise levels.

Supporting Information Text S4: PLD optimization for 3D acquisitions

We re-ran the CBF-ATT$_{opt}$ and CBF$_{opt}$ optimizations, described in the methods section of the article, but this time assuming a single excitation pulse (appropriate for 3D readouts or single-slice acquisitions). A readout duration of 500 ms was assumed due to the necessarily shorter readout durations of 3D methods. The number of PLDs were kept to ≤ 10 to enable their use with segmented acquisitions. Two uniform ATT distributions were used to produce optimal protocols: a healthy ATT range of $0.5 \leq ATT \leq 2$ s and a prolonged ATT range of $1 \leq ATT \leq 3$ s. The healthy range was extended to 2 s because we found a small percentage of GM voxels in our in vivo ASL data had ATTs longer than 1.8 s. Both distributions had 0.3 s linearly decreasing tapers at the edges of the distribution to avoid edge effects. We kept the label duration fixed at 1.4 s for consistency with the other protocols in this work. All other optimization parameters were the same as those described in the methods.

The optimized PLDs are given *Supporting Information Table S1* and shown in *Supporting Information Figure S9*, where they are compared to protocols with evenly distributed PLDs across the ATT ranges. The number of averages assumes a single-shot acquisition. The protocol timings are identical for segmented acquisitions (where the segment number is a factor of the number of averages), but with the number of averages being divided by the number of segments used (ignoring small differences in acquisition time), with an associated increase of the CRLBs by the same amount (ignoring differences in TE).

The overall RMSEs for the standard ATT range were: Even (CBF = 5.5 ml/100g/min, ATT = 0.12 s), CBF-ATT$_{opt}$ (CBF = 5.2 ml/100g/min, ATT = 0.09 s) and CBF$_{opt}$ (CBF = 4.7 ml/100g/min, ATT = 0.13 s). The overall RMSEs for the prolonged ATT range were: Even (CBF = 12.9 ml/100g/min, ATT = 0.27 s), CBF-ATT$_{opt}$ (CBF = 9.9 ml/100g/min, ATT = 0.24 s) and CBF$_{opt}$ (CBF = 9.9 ml/100g/min, ATT = 0.29 s).

For both ATT distributions, the optimal protocols had reduced RMSE variation across the ATT distribution and achieved their respective aims of minimizing both CBF and ATT errors or just minimizing CBF errors across the ATT range. It should be noted that the errors in the prolonged ATT distribution are quite large for all the protocols. Further improvement in CBF and ATT accuracy in the presence of delayed ATT may be achieved by also optimizing the label duration.

Supporting Information Table S1: Optimal PLDs for 3D acquisitions

Protocol	*Post-labeling delays (s)*	*# PLDs*	*# Averages*
$\mathbf{0.5 \leq \mathit{ATT} \leq 2}$ **s**			
Even	0.5, 0.725, 0.925, 1.15, 1.35, 1.575, 1.775, 2	8	6
CBF-ATT_{opt}	0.2, 0.9, 0.9, 1.15, 2, 2.2	6	8
CBF_{opt}	0.2, 0.975, 1.425, 1.85, 2.025, 2.15, 2.25, 2.3, 2.3, 2.3	10	4
$\mathbf{1 \leq \mathit{ATT} \leq 3}$ **s**			
Even	1, 1.25, 1.5, 1.75, 2, 2.25, 2.5, 2.75, 3	9	4
CBF-ATT_{opt}	0.7, 1.875, 1.9, 1.9, 1.925, 2.75, 2.95, 3.1, 3.3	9	4
CBF_{opt}	0.7, 1.8, 2.5, 2.875, 3.075, 3.3, 3.3, 3.325	8	4

Supporting Information Figure S1

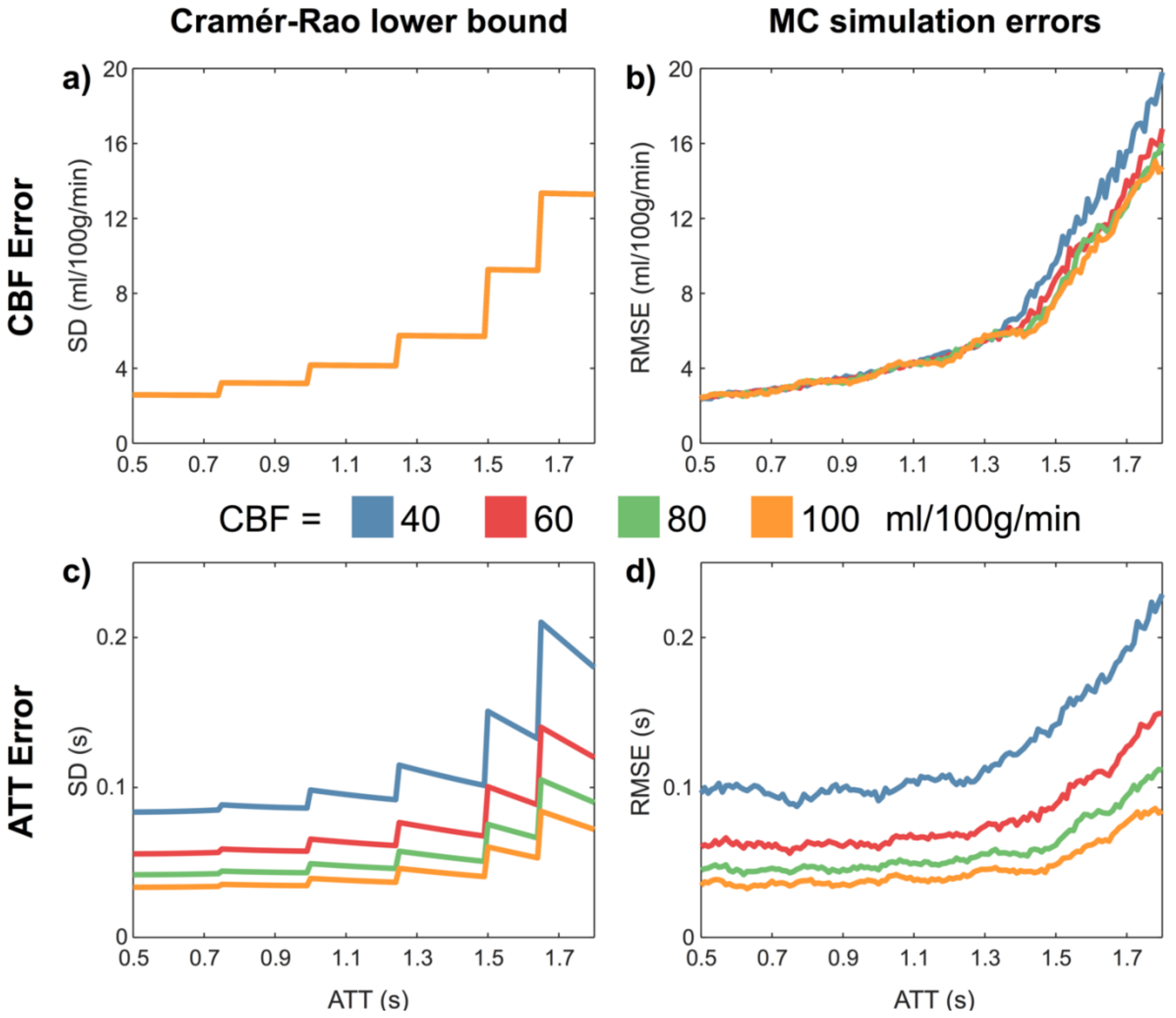

Supporting Information Figure S1:

The effect of true CBF on estimation errors in CBF (top row) and ATT (bottom row). Both the predicted Cramér-Rao lower bound (a, c) and RMSEs for Monte Carlo simulations (b, d) are shown.

These results demonstrate that the CBF estimation errors do not vary greatly with CBF, while the ATT errors are inversely proportional to CBF. The reference multi-PLD protocol for one slice was used for this demonstration.

__Supporting Information Figure S2__

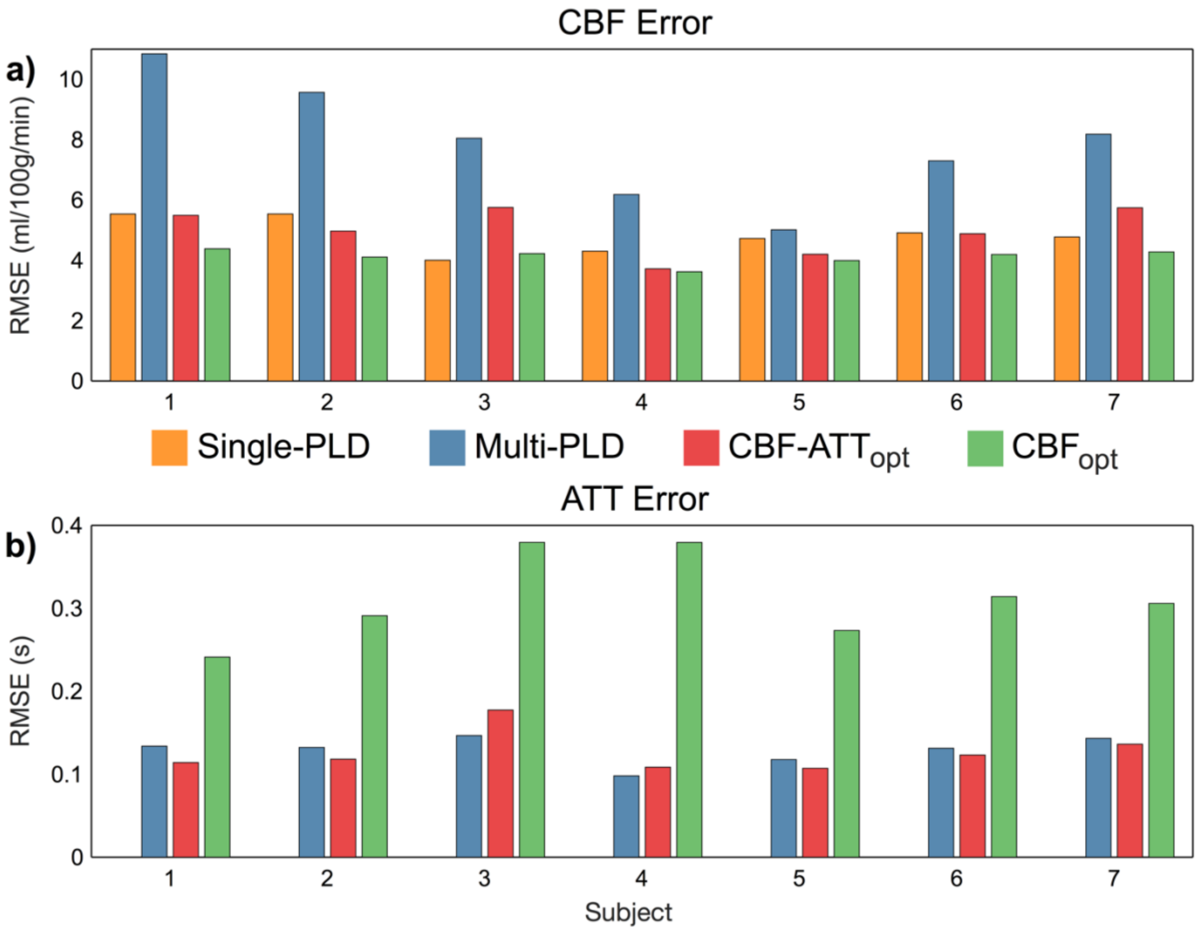

Supporting Information Figure S2:

In vivo CBF (a) and ATT (b) RMSEs for each of the 7 subjects. The trends are extremely similar across the subjects, demonstrating the robustness of the optimization.

Supporting Information Figure S3

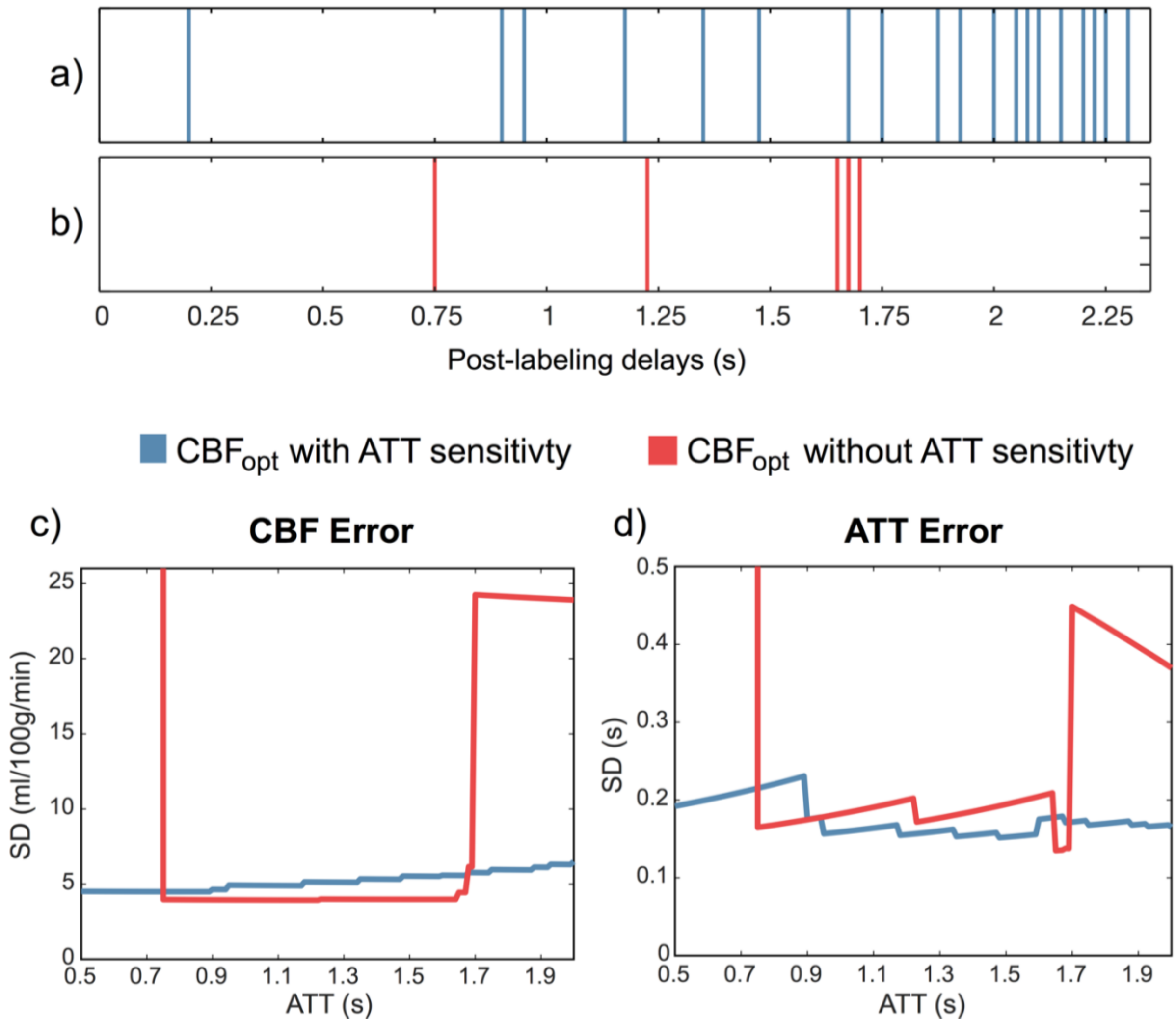

Supporting Information Figure S3:

The PLDs (a, b) and the predicted CBF and ATT errors (Cramér-Rao lower bound (CRLB) SD) (c, d) for CBF_{opt} with and without the ATT sensitivity function in the FIM. We assumed a 0.5 s readout duration for a 3D acquisition, 28 PLDs and an ATT range of 0.5 – 2 s (similar to Supporting Information Text S4). Repeated PLDs are not shown. CBF and ATT CRLBs at ATTs shorter than 0.75 s are extremely large for the protocol without the ATT sensitivity function included and are out of view for clarity.

The differences in the chosen PLDs and the resulting CRLBs demonstrate the importance of including the ATT sensitivity function in the FIM. If the ATT sensitivity function is not included in the FIM, then it is implicitly assumed that the ATT is known, which can result in large errors.

Supporting Information Figure S4

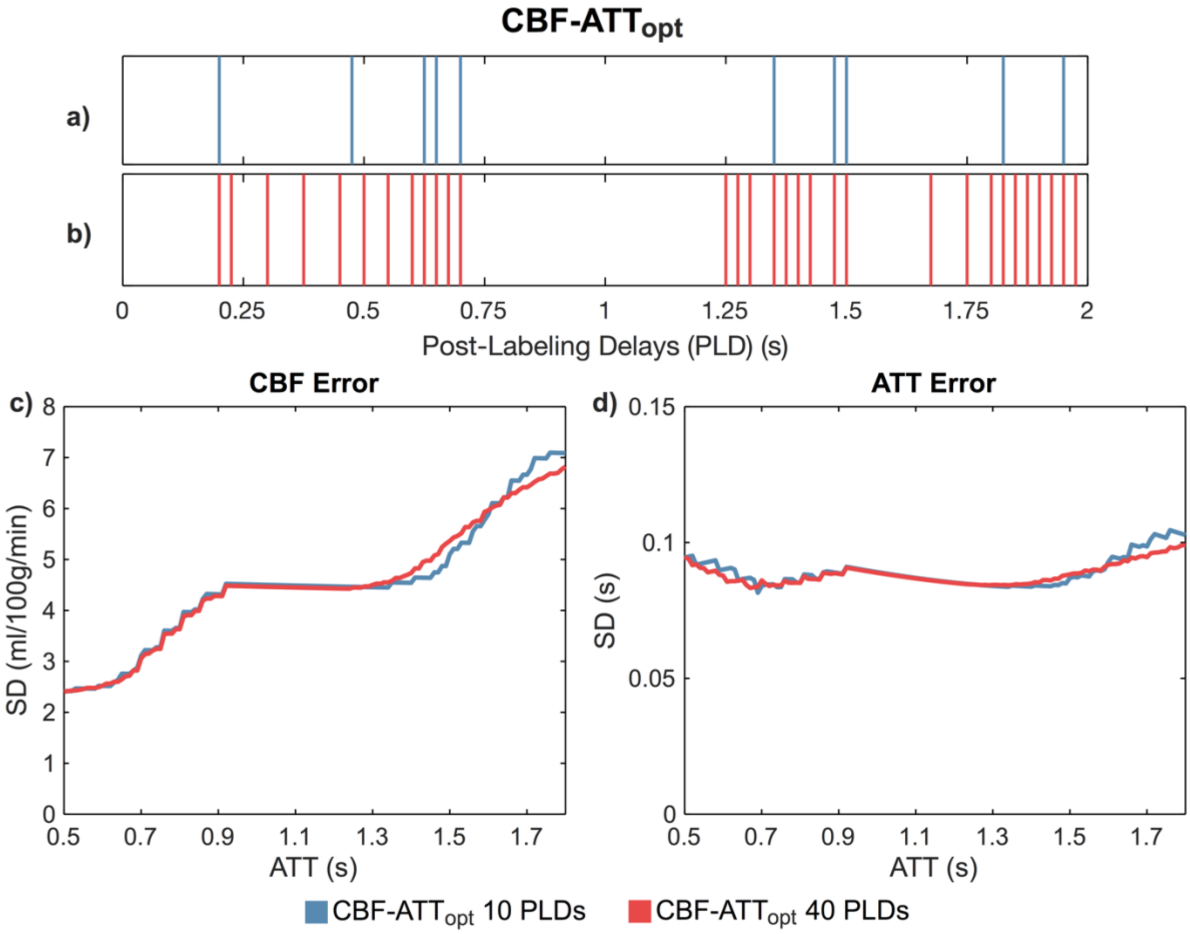

Supporting Information Figure S4:

The PLDs (a, b) and the predicted CBF and ATT errors (Cramér-Rao lower bound (CRLB) SD) (c, d) for the CBF-ATT$_{opt}$ protocol restricted to either 10 or 40 PLDs. The 10 PLD protocol uses 4 averages of the 10 PLDs, while the 40 PLD protocol only has one average for each PLD. Using 10 PLDs rather than 40 PLDs only resulted in an average ~0.5% and ~1% increase in the CBF and ATT CRLBs, respectively.

Supporting Information Figure S5

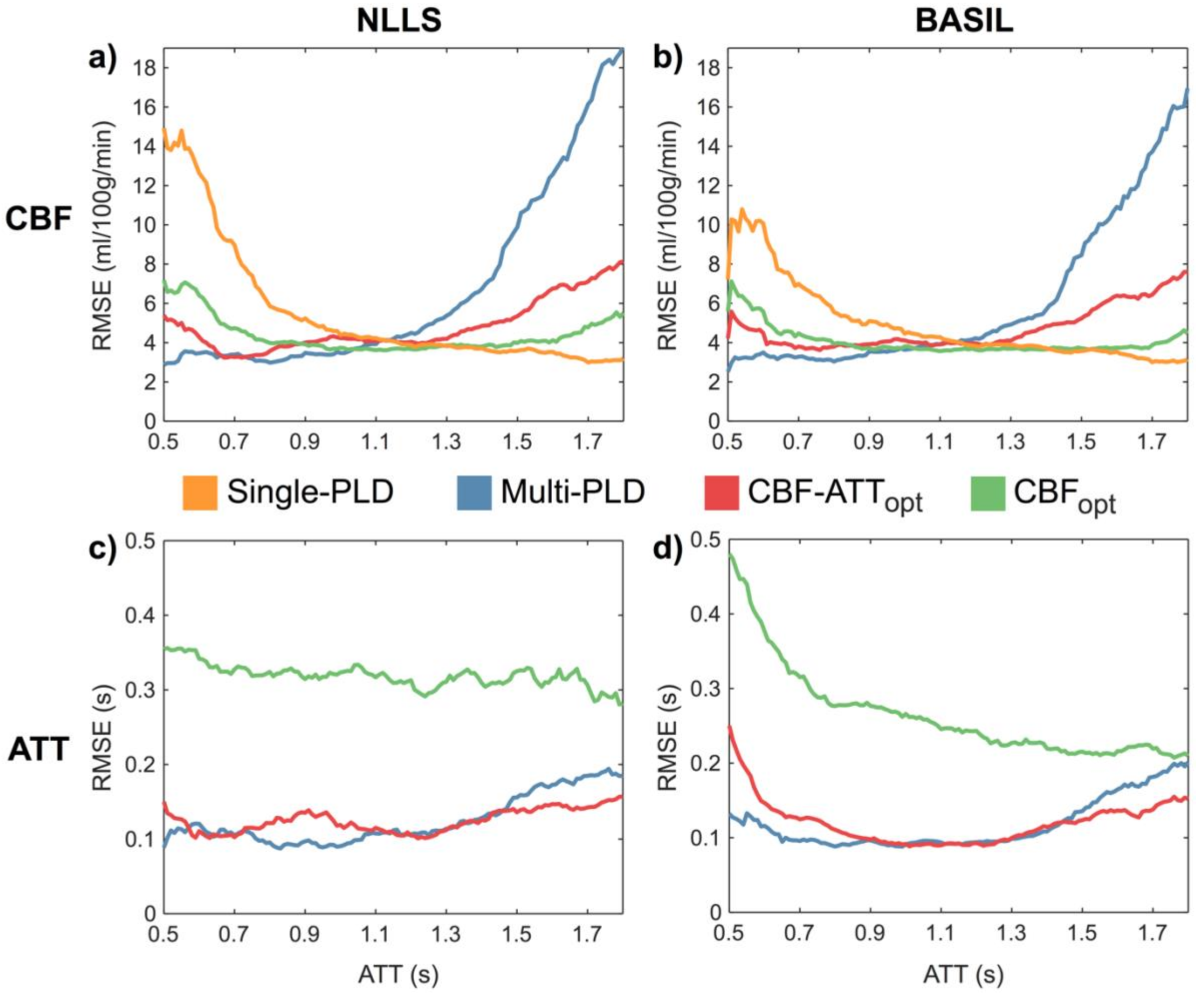

Supporting Information Figure S5:

In vivo RMSEs of CBF (top row) and ATT (bottom row) for data fitted with the NLLS method, as in Figure 6 (b, d), and with BASIL[29] (b, d), a variational Bayesian algorithm. The fitting priors used for the BASIL fitting were (mean ± SD): 0 ± 10^6 ml/100g/min and 1.25 ± 1 s, for CBF and ATT respectively.

Similarly to the NLLS data, only BASIL fitted data which had CBF and ATT posterior distribution SD less than 5 ml/100g/min and 0.1 s, respectively, were included in the graph. BASIL reduced CBF errors, particularly in regions with very large errors in the NLLS fitting. Mean ATT errors were also reduced, but were larger at short ATTs. This suggests that BASIL produces better CBF estimates from noisy data than a naïve NLLS algorithm, but there remain significant benefits from appropriately optimizing the PLDs in both cases.

Supporting Information Figure S6

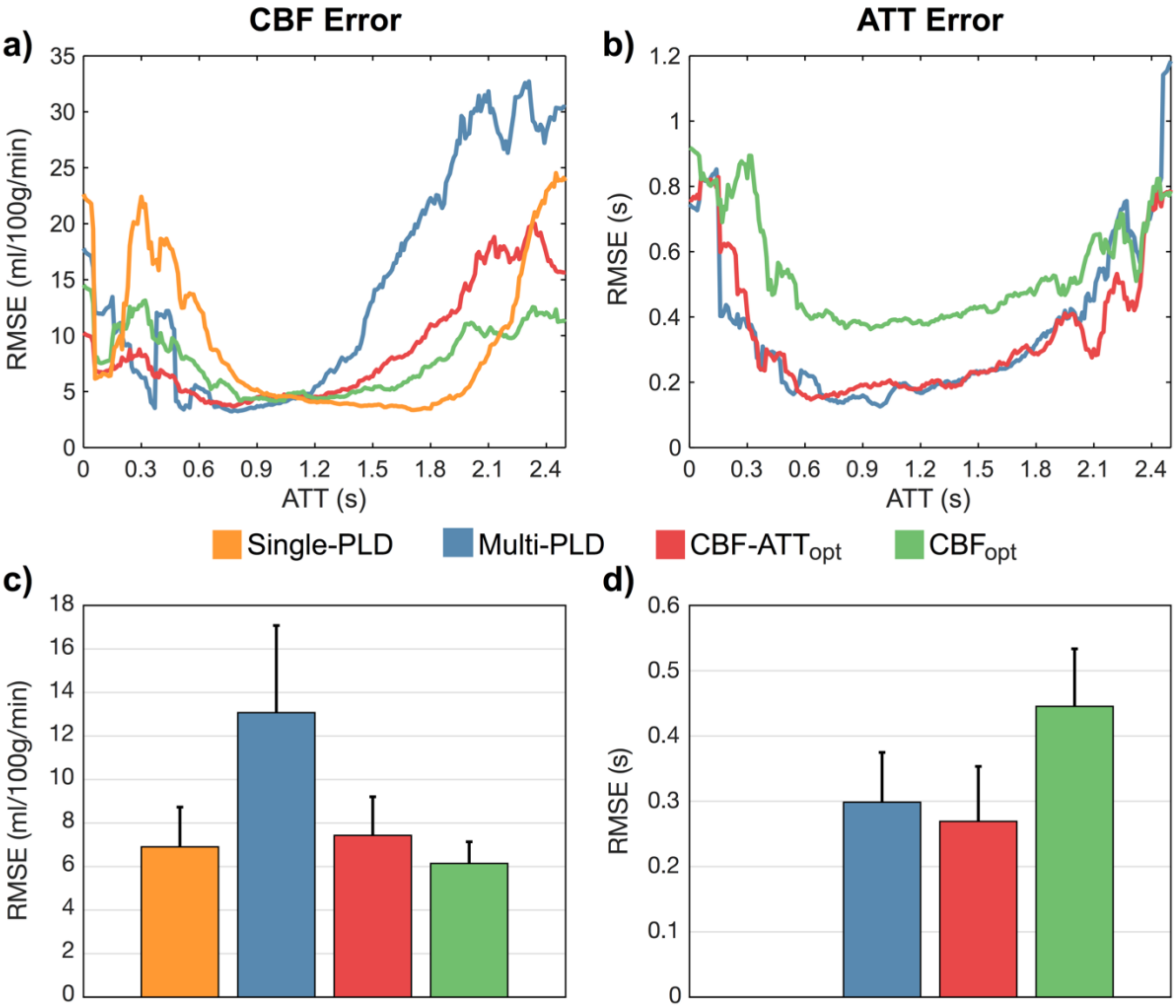

Supporting Information Figure S6:

In vivo RMSEs of CBF (left column) and ATT (right column) estimates for all GM data for the 7 subjects with no other data exclusion criteria imposed. The top row (a, b) shows the RMSE trends across ATTs, while the bottom row (c, d) shows the mean and SD of the RMSEs across subjects. The general trends remain unchanged, compared to Figure 6 and Figure 7.

As expected, there is a general increase in RMSEs compared to Figure 6 and Figure 7 due to the removal of the ground truth data exclusion criteria. Graphs a) and b) agree well with Figure 6 in the ATT prior range of $0.5 \leq ATT \leq 1.8$. The trends in c) and d) also agree well with Figure 7, though the CBF_{opt} and single-PLD CBF RMSEs are no longer significantly different. This is to be expected since there is greater noise in the ground truth estimates and data from outside the optimized range has been included.

Supporting Information Figure S7

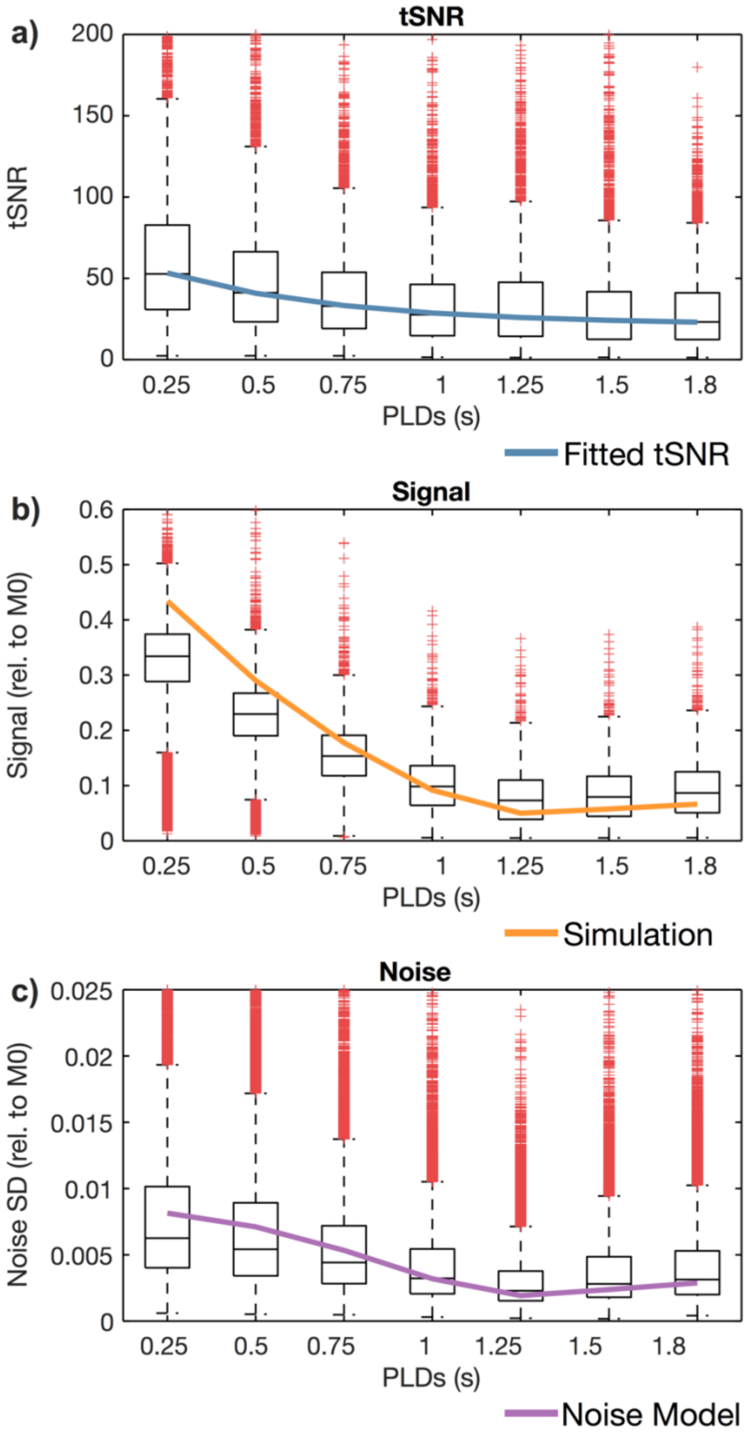

Supporting Information Figure S7:

Boxplots of the tSNR, signal and noise of the GM voxels from the control ASL data of the reference multi-PLD and single-PLD data for all 7 subjects.

The median tSNR has been fit using an exponential decay model, while the tissue signal (normalized by M_{0B}) was simulated using a series of saturation and inversion recovery models. The noise can then be modeled as the simulated signal divided by the fitted tSNR.

Supporting Information Figure S8

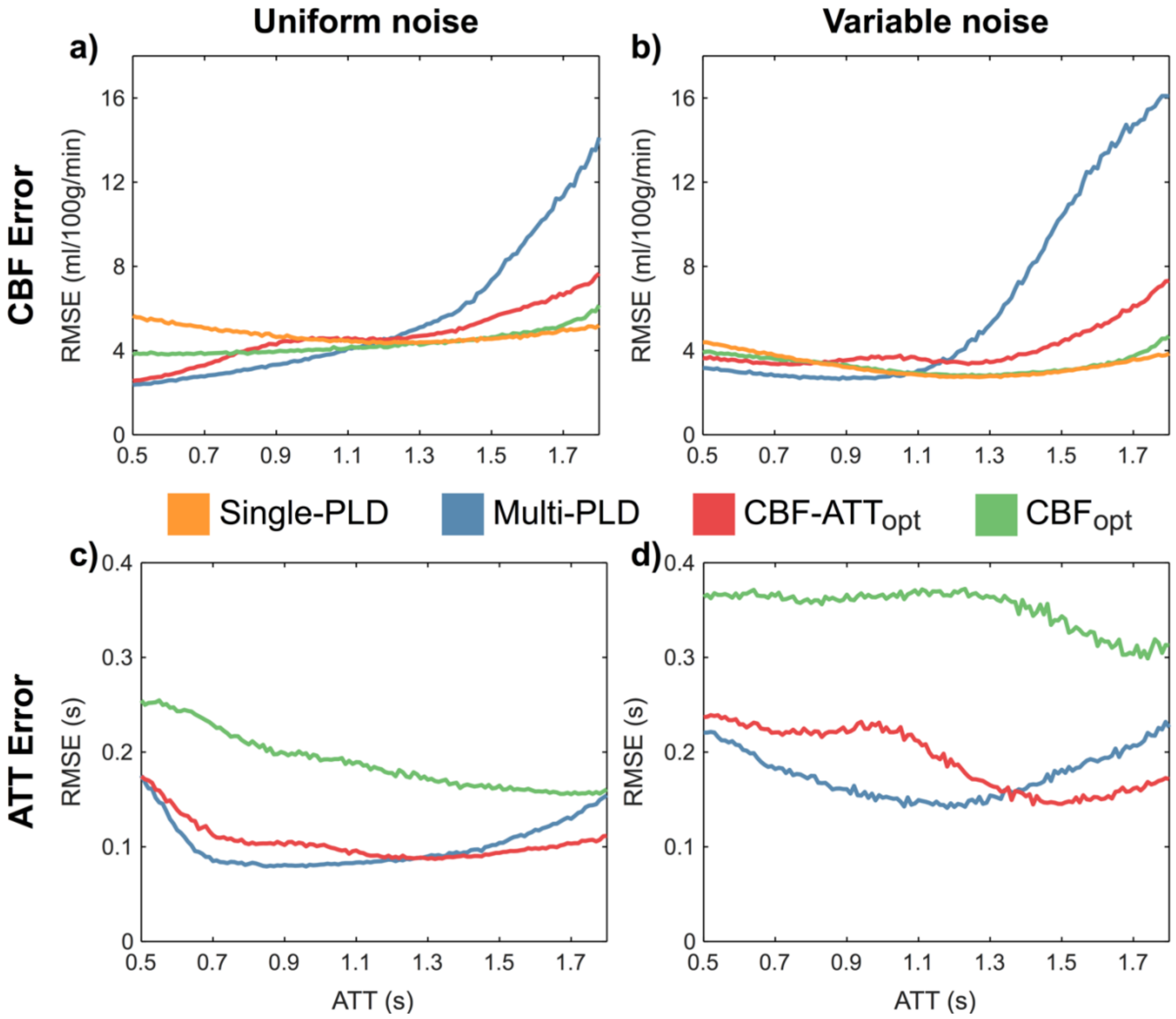

Supporting Information Figure S8:

The effect on CBF (a, b) and ATT (c, d) RMSEs when using uniform (a, c) or variable (b, d) noise across PLDs. Details of the simulations are given in Supporting Information Text S3.

The broad trends are consistent. However, all protocols except the reference multi-PLD protocol have reduced CBF errors at late ATTs and the ATT errors for all protocols increased across the entire ATT range. The single-PLD, CBF-ATT$_{opt}$ and CBF$_{opt}$ CBF estimates benefit from having many PLDs at times of reduced noise with the variable noise model.

Supporting Information Figure S9

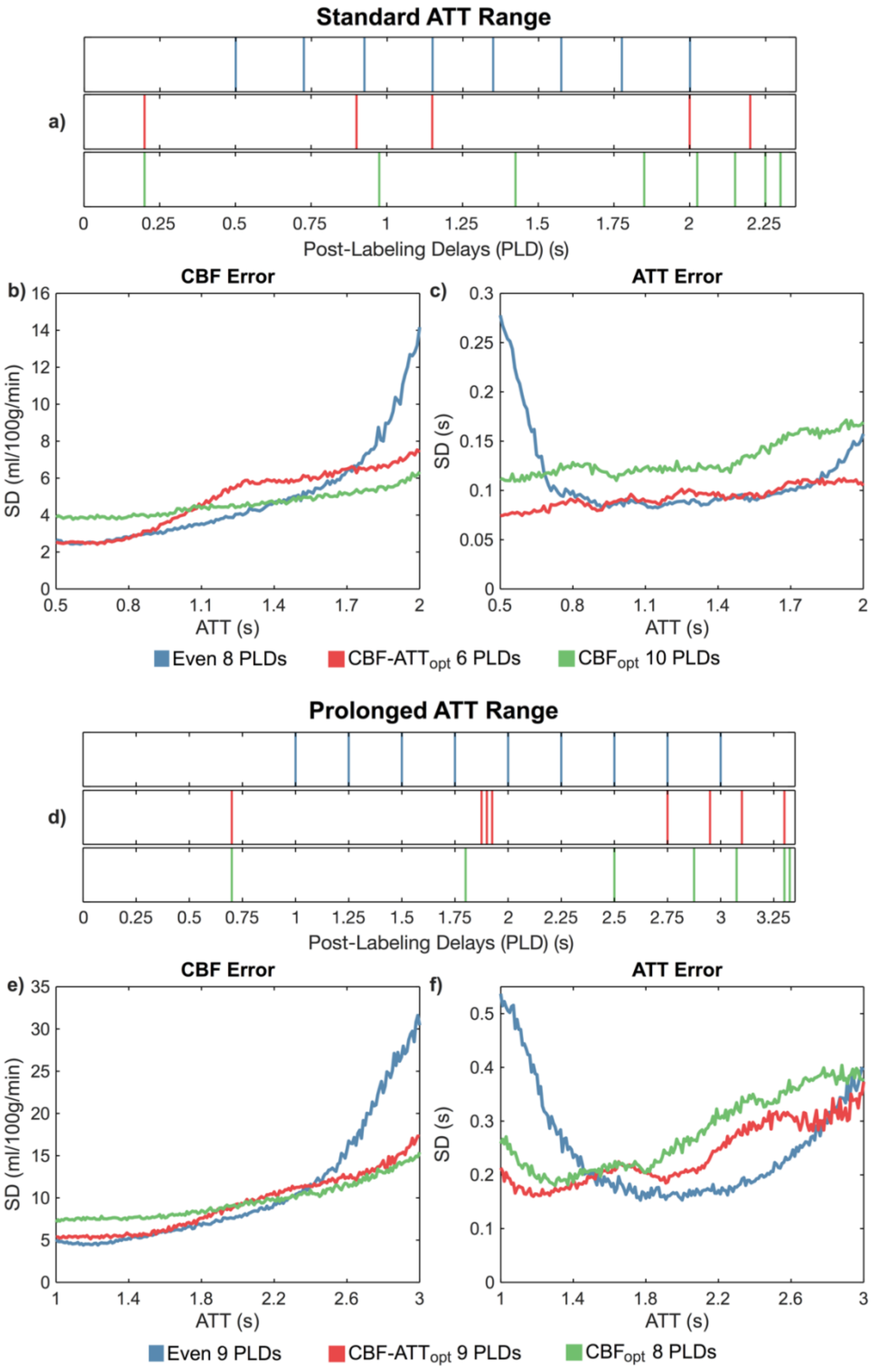

Supporting Information Figure S9:

The PLDs (a, d) and the MC simulation RMSEs (b, c, e, f) for Even, CBF-ATT$_{opt}$ and CBF$_{opt}$ protocols over a standard healthy range of ATTs ($0.5 \leq ATT \leq 2$) s and a prolonged ATT range ($1 \leq ATT \leq 3$). The timings shown are for a 3D acquisition. Repeated PLDs are not shown, but are listed in full in Supporting Information Table S1.
